# FK506 regulates Ca^2+^ release evoked by inositol 1,4,5‐trisphosphate independently of FK‐binding protein in endothelial cells

**DOI:** 10.1111/bph.14905

**Published:** 2020-01-26

**Authors:** Charlotte Buckley, Calum Wilson, John G. McCarron

**Affiliations:** ^1^ Strathclyde Institute of Pharmacy and Biomedical Science University of Strathclyde Glasgow UK

## Abstract

**Background and Purpose:**

FK506 and rapamycin are modulators of FK‐binding proteins (FKBP) that are used to suppress immune function after organ and hematopoietic stem cell transplantations. The drugs share the unwanted side‐effect of evoking hypertension that is associated with reduced endothelial function and nitric oxide production. The underlying mechanisms are not understood. FKBP may regulate IP_3_ receptors (IP_3_R) and ryanodine receptors (RyR) to alter Ca^2+^ signalling in endothelial cells.

**Experimental Approach:**

We investigated the effects of FK506 and rapamycin on Ca^2+^ release via IP_3_R and RyR in hundreds of endothelial cells, using the indicator Cal‐520, in intact mesenteric arteries from male Sprague‐Dawley rats. IP_3_Rs were activated by acetylcholine or localised photo‐uncaging of IP_3_, and RyR by caffeine.

**Key Results:**

While FKBPs were present, FKBP modulation with rapamycin did not alter IP_3_‐evoked Ca^2+^ release. Conversely, FK506, which modulates FKBP and blocks calcineurin, increased IP_3_‐evoked Ca^2+^ release. Inhibition of calcineurin (okadiac acid or cypermethrin) also increased IP_3_‐evoked Ca^2+^ release and blocked FK506 effects. When calcineurin was inhibited, FK506 reduced IP_3_‐evoked Ca^2+^ release. These findings suggest that IP_3_‐evoked Ca^2+^ release is not modulated by FKBP, but by FK506‐mediated calcineurin inhibition. The RyR modulators caffeine and ryanodine failed to alter Ca^2+^ signalling suggesting that RyR is not functional in native endothelium.

**Conclusion and Implications:**

The hypertensive effects of the immunosuppressant drugs FK506 and rapamycin, while mediated by endothelial cells, do not appear to be exerted at the documented cellular targets of Ca^2+^ release and altered FKBP binding to IP_3_ and RyR.

**Abbreviations**
2‐APB: 2‐aminoethoxydiphenyl borate
*F*: fluorescence intensity
*F*_0_: baseline fluorescence intensity
FKBPs: FK‐binding proteins
IP_3_: inositol 1,4,5‐trisphosphate
IP_3_R: inositol 1,4,5‐trisphosphate receptors
RyR: ryanodine receptors

**What is already known**

- FK506 and rapamycin modulate FK‐binding proteins (FKBP) but share the side effect of evoking hypertension.
- Hypertension induced by FK506 and rapamycin is associated with reduced endothelial function and NO production.

**What this study adds**

- FK506 increases IP_3_‐evoked Ca^2+^ release by calcineurin inhibition; rapamycin does not increase IP_3_‐evoked Ca^2+^ release.
- RyR activation failed to evoke Ca^2+^ release, and ryanodine does not alter endothelial Ca^2+^ signals.

**What is the clinical significance**

- The hypertensive effects of FK506 and rapamycin are not FKBP‐mediated in endothelial cells.
- IP_3_‐mediated Ca^2+^ release may be modulated indirectly by FK506 through inhibition of calcineurin.

## 1 INTRODUCTION

https://www.guidetopharmacology.org/GRAC/LigandDisplayForward?ligandId=6784 (also known as tacrolimus) and https://www.guidetopharmacology.org/GRAC/LigandDisplayForward?ligandId=6031 (sirolimus) are immunosuppressant drugs that are used widely in organ transplantation to prevent tissue rejection (Jeanmart et al., 2002; Lemos et al., 2003; Wallemacq et al., 2009; Wallemacq & Reding, 1993). Rapamycin is also used in drug‐eluting stents to prevent re‐stenosis in patients with coronary artery disease (Abizaid, 2007; Serruys, Regar, & Carter, 2002). While the drugs are effective immunosuppressants, FK506 and rapamycin have the undesirable effect of causing hypertension in up to 70% of patients that are treated with the drugs (Lindenfeld et al., 2004; Lindenfeld et al., 2005; Taylor et al., 1999). The underlying causes of this immunosuppressant‐induced hypertension are not understood, but disruption of normal endothelial function and decreased production of the endogenous vasodilator https://www.guidetopharmacology.org/GRAC/LigandDisplayForward?ligandId=2509 are reported contributors (De Lima et al., 1999; Takeda, Miyamori, Furukawa, Inaba, & Mabuchi, 1999).

Endothelial function and NO production are critically controlled by the cytoplasmic Ca^2+^ concentration. In turn, endothelium‐derived Ca^2+^‐dependent mediators control vascular tone, nutrient exchange, blood cell recruitment, blood clotting, and the formation of new blood vessels (see Feletou, 2011). The endoplasmic reticulum (ER), the intracellular Ca^2+^ store, is of particular importance in regulating the cytoplasmic Ca^2+^ concentration in endothelial cells (Tran & Watanabe, 2006) from which release is controlled by two major channel receptor complexes—the inositol 1,4,5‐trisphosphate receptors (https://www.guidetopharmacology.org/GRAC/FamilyDisplayForward?familyId=123) and ryanodine receptors (https://www.guidetopharmacology.org/GRAC/FamilyDisplayForward?familyId=125). While the contribution of IP_3_R is of acknowledged significance in regulating Ca^2+^, the role of RyR in endothelial cells in intact arteries is less certain.

The cytoplasmic receptors for the immunosuppressant drugs FK506 and rapamycin have been identified as the FK‐binding proteins (https://www.guidetopharmacology.org/GRAC/ObjectDisplayForward?objectId=2609s; Bierer et al., 1990; Harding, Galat, Uehling, & Schreiber, 1989). FKBPs are accessory proteins of IP_3_R and RyR on the internal Ca^2+^ store. The association of FKBP with the IP_3_R and RyR is disrupted by FK506 and rapamycin and may change channel activity with consequences for Ca^2+^ signalling (Bultynck, De Smet, et al., 2001; Cameron, Steiner, Sabatini, et al., 1995; Dargan, Lea, & Dawson, 2002). Indeed, changes in the association of FKBPs with the channels may evoke significant effects on cardiovascular function. Genetic deletion of FKBP12.6 in mice causes hypertension (Long, Cook, Wu, & Mitchell, 2007). In rats, FK506 decreased production of the endothelium‐derived vasodilator NO and increased the production of the contractile agent endothelin (Takeda et al., 1999). In human and rat resistance arteries in vitro, FK506 increased the sensitivity to https://www.guidetopharmacology.org/GRAC/LigandDisplayForward?ligandId=484 and impaired vasodilation to https://www.guidetopharmacology.org/GRAC/LigandDisplayForward?ligandId=294 and https://www.guidetopharmacology.org/GRAC/LigandDisplayForward?ligandId=9533 (De Lima et al., 1999). Reduced endothelium‐dependent relaxation was also reported in mouse aorta after FK506 treatment (Chiasson et al., 2011; Cook, Chiasson, Long, Wu, & Mitchell, 2009). These findings raise the possibility that IP_3_R and RyR may contribute to the altered endothelial function induced by the immunosuppressant drugs. However, the precise nature of the control that FKBP might exert on IP_3_R and RyR is uncertain.

In the case of IP_3_R, some investigations have found removal of FKBP12 decreased https://www.guidetopharmacology.org/GRAC/LigandDisplayForward?ligandId=4222‐mediated Ca^2+^ release (MacMillan, Currie, Bradley, Muir, & McCarron, 2005), while (in bilayer studies) addition of exogenous FKBP12 increased IP_3_R channel activity (Dargan et al., 2002). These results suggest that FKBP12 *potentiates* IP_3_R activity. On the other hand, FKBP12 may *inhibit* channel activity, and removal of FKBP12 from the channel increased IP_3_R‐mediated Ca^2+^ release in rat cerebral microsomes and smooth muscle (Cameron, Steiner, Sabatini, et al., 1995). Despite the importance of IP_3_‐mediated Ca^2+^ release to the control of endothelial function, there are no investigations that have examined FKBP regulation of IP_3_‐mediated Ca^2+^ release in endothelial cells.

FKBP12 may also associate with RyR (Bradley, Currie, MacMillan, Muir, & McCarron, 2003; Bultynck, De Smet, et al., 2001; Carmody, Mackrill, Sorrentino, & O'Neill, 2001; MacMillan et al., 2005; MacMillan, Currie, & McCarron, 2008; Tang, Chen, Zou, Campbell, & Li, 2002; Wang et al., 2004; Zheng et al., 2004). Removal of FKBPs from RyR by either FK506 or rapamycin increased RyR channel open probability in lipid bilayers (Kaftan, Marks, & Ehrlich, 1996; Tang et al., 2002) and Ca^2+^ signals in intestinal, colonic, bladder, and pulmonary artery myocytes (Bielefeldt, Sharma, Whiteis, Yedidag, & Abboud, 1997; MacMillan et al., 2008; Weidelt & Isenberg, 2000; Zheng et al., 2004). In mesenteric and human small resistance arteries, FK506 induced vasoconstriction (De Lima et al., 1999; Schwertfeger, Wehrens, Oberhauser, Katzenwadel, & Rump, 2001), while in rat vas deferens, rapamycin decreased phenylephrine‐induced contractions as a result of Ca^2+^ leak via RyR (Scaramello, Muzi‐Filho, Zapata‐Sudo, Sudo, & Cunha Vdo, 2009). FKBP12.6 deficient mice showed increased spontaneous Ca^2+^ release from the internal store when compared with wild type urinary bladder myocytes (Ji et al., 2004). Rebinding either FKBP12 or FKBP12.6, following their removal, decreased channel opening (Barg, Copello, & Fleischer, 1997; Brillantes et al., 1994; Bultynck, Rossi, et al., 2001; Mayrleitner, Timerman, Wiederrecht, & Fleischer, 1994; Timerman et al., 1993). There are also reports of Ca^2+^ release via RyR being altered by FKBP in endothelial cells. In cultured mouse aortic endothelial cells depletion of FKBP increased endothelial intracellular Ca^2+^ leak via RyR, suggesting that FKBP stabilized the channel in the closed state (Cook et al., 2009; Long et al., 2007). Furthermore, rapamycin or FK506 decreased NO production and endothelium‐dependent dilation and increased systolic BP (Long et al., 2007).

However, evidence does not universally support a role of FKBPs in regulating either RyR or IP_3_R activity. In some studies, no interaction was found to occur between FKBP and either RyR (Carmody et al., 2001; Murayama et al., 1999; Wang et al., 2004; Zheng et al., 2004) or IP_3_R (Bultynck, De Smet, et al., 2001; Carmody et al., 2001; Thrower et al., 2000; Zheng et al., 2004). Functional studies also failed to detect any effect of the drug FK506 or protein FKBP on IP_3_‐mediated Ca^2+^ release (Boehning & Joseph, 2000; Bultynck, De Smet, et al., 2001; Bultynck et al., 2000; Kanoh et al., 1999) or RyR channel function (Barg et al., 1997; duBell, Wright, Lederer, & Rogers, 1997; Epstein, Beall, Wynn, Mulloy, & Brophy, 1998; Timerman et al., 1996; Xiao et al., 2007; Yasutsune et al., 1999).

In addition to binding to the IP_3_R and RyR, FKBPs may regulate kinase and phosphatase activity. The different modes of action of separate signalling pathways may account, in part, for the contradictory findings on IP_3_R and RyR modulation by FKBP. Removal of FKBP from either IP_3_R or RyR by FK506 results in the formation of a FK506‐FKBP complex, which inhibits the Ca^2+^/calmodulin‐dependent serine/threonine phosphatase calcineurin (Liu et al., 1991). Calcineurin may mediate the immunosuppressive actions of FK506 (Liu et al., 1992), contribute to various cellular functions (Guerini, 1997; Klee, Ren, & Wang, 1998), and regulate RyR and IP_3_R to control Ca^2+^ release (Bandyopadhyay, Shin, Ahn, & Kim, 2000; Cameron et al., 1997; Cameron, Steiner, Roskams, et al., 1995; Cameron, Steiner, Sabatini, et al., 1995; MacMillan et al., 2008; Shin et al., 2002). In support, calcineurin inhibitors increased https://www.guidetopharmacology.org/GRAC/LigandDisplayForward?ligandId=407‐ and ryanodine‐induced Ca^2+^ release and the frequency and amplitude of Ca^2+^ oscillations in cardiac and skeletal muscle (Bandyopadhyay, Shin, Ahn, & Kim, 2000; Shin et al., 2002). In COS‐7 cells and cerebellar microsomes, calcineurin inhibition increased ATP‐induced Ca^2+^ release and IP_3_R activity (Bandyopadhyay, Shin, & Kim, 2000; Cameron, Steiner, Roskams, et al., 1995) whereas expression of a constitutively active form of calcineurin inhibited Ca^2+^ release (Bandyopadhyay, Shin, & Kim, 2000). Conversely, other studies have shown that drugs that inhibit calcineurin, such as cypermethrin, cyclosporin A, or okadaic acid, did not alter Ca^2+^ release via either RyR or IP_3_R (Ashizawa, Kobayashi, Tanaka, & Nakayama, 1989; Avdonin et al., 1999; Cook et al., 2009; Frapier et al., 2001; Hirano, Kanaide, & Nakamura, 1989; Macmillan & McCarron, 2009; Zheng et al., 2004). The comparatively few studies in endothelial cells also yielded contradictory findings. In cultured endothelial cells (ECV304, BPAECs, and HPAECs), the calcineurin inhibitors okadaic acid, cyclosporin A, or calyculin A evoked Ca^2+^ release from the ER (Hepworth, Lawrie, & Simpson, 1997; Kolozsvari et al., 2012). However, calcineurin activity failed to alter Ca^2+^ in cultured aortic endothelial cells (Cook et al., 2009). Together, these studies highlight a confused picture of the regulation of endothelial Ca^2+^ signalling by FKBP and its disruption via the immunosuppressant drugs FK506 and rapamycin.

The present study was undertaken in view of the controversy which surrounds the effects of the drugs FK506 and rapamycin on intracellular Ca^2+^ release, their importance as immunosuppressant drugs, associated hypertension side effects, and the absence of information on the role of FKBP on IP_3_‐mediated Ca^2+^ release. Ca^2+^ was measured in endothelial cells of intact mesenteric arteries. Around 100 cells were analysed separately from each preparation and cellular responses before and after various pharmacological interventions were paired. The use of photolysed caged IP_3_ to activate IP_3_R minimized the number of second messenger systems activated. The study has shown that pharmacological modulation of FKBP from IP_3_R with FK506 increased IP_3_‐evoked Ca^2+^ release. Cypermethrin and https://www.guidetopharmacology.org/GRAC/LigandDisplayForward?ligandId=5349 (drugs which can inhibit calcineurin) each increased IP_3_‐evoked Ca^2+^ release and prevented the FK506‐induced increase in Ca^2+^ release. Rapamycin, which removes FKBP from the receptors but does not inhibit calcineurin, did not alter IP_3_‐evoked Ca^2+^ release. Thus, calcineurin is required for the potentiation of IP_3_‐evoked Ca^2+^ release by FK506. The RyR activator caffeine did not evoke Ca^2+^ release in the endothelium in intact arteries, suggesting that RyR does not play a role in endothelial Ca^2+^ modulation. Our results suggest that IP_3_R‐mediated Ca^2+^ release may be modulated (a) indirectly by FKBP through inhibition of calcineurin, and (b) FKBPs by themselves do not appear to regulate Ca^2+^ release in endothelial cells.

## 2 METHODS

### 2.1 Animals

All animal husbandry and experimental procedures were carried out in accordance with the prior approval of the University of Strathclyde Animal Welfare and Ethical Review Body and under relevant UK Home Office Regulations (Schedule 1 of the Animals [Scientific Procedures] Act 1986, UK). Strathclyde BPU is a conventional unit which undertakes FELASA quarterly heath monitoring. Male Sprague–Dawley rats (10–12 week old; 250–300 g), from an in‐house colony, were used for the study. The animals were housed three per cage, and the cage type was North Kent Plastic model RC2F with nesting material “Sizzle Nest.” A 12:12 light dark cycle was used with a temperature range of 19–23°C (set point 21°C) and humidity levels between 45% and 65%. Animals had free access to fresh water and SDS diet RM1 (rodent maintenance). The enrichment in the cages was aspen wood chew sticks and hanging huts. Animal studies are reported in compliance with the ARRIVE guidelines (Kilkenny, Browne, Cuthill, Emerson, & Altman, 2010) and with the recommendations made by the British Journal of Pharmacology (McGrath & Lilley, 2015).

All experiments used either the aorta or second‐order mesenteric arteries (as described) obtained from male Sprague–Dawley rats (10–12 weeks old; 250–350 g), killed by an overdose of pentobarbital sodium (200 mg·kg^−1^, i.p.). Sprague–Dawley rats are a widely used experimental model with a wealth of background information to aid interpretation of results. Controls and experimental treatments were carried out in the same tissue, so blinding and randomization were not used.

### 2.2 Mesenteric artery preparation and mounting

Arteries were prepared as previously described (Wilson, Lee, & McCarron, 2016). In brief, the mesenteric bed was removed immediately following killing. Second‐order mesenteric arteries were dissected and opened longitudinally using microscissors. The arteries were pinned out on a Sylgard block using 50‐μm diameter wire with the endothelial layer exposed. Throughout this procedure, preparations were kept in physiological salt solution (PSS; 145‐mM NaCl, 2‐mM MOPS, 4.7‐mM KCl, 1.2‐mM NaH_2_PO_4_, 5‐mM glucose, 0.02‐mM EDTA, 1.17‐mM MgCl_2_, 2‐mM CaCl_2_, pH 7.4). Arteries were then incubated with the Ca^2+^ indicator Cal‐520 (5 μM) with 0.02% Pluronic F‐127 for 30 min at 37°C. After washing in PSS, preparations were placed face down on 200‐μm diameter stainless steel pins on a 0 grade thickness coverslip that was fixed to the bottom of a custom bath chamber (3 cm × 1.5 cm; Wilson, Lee, & McCarron, 2016).

### 2.3 Mesenteric artery image acquisition and analysis

Images were acquired using a Nikon Eclipse TE300 inverted microscope body with a 40× 1.3 NA Nikon S Fluor oil‐immersion objective lens, imaged onto an Andor iXon EMCCD camera, cropped to 512 × 512 pixels. A CoolLED pE‐300 LED system with 405/488/561 nm excitation light was used in conjunction with custom‐designed FITC/TRITC excitation and emission filters and beamsplitter. All acquisition was performed through MicroManager v1.4.22 (Edelstein et al., 2014).

### 2.4 Localized IP_3_ uncaging

A Rapp Optoelectronics flash lamp (00‐325‐JML‐C2) used at 200 V produced light of ~1‐ms duration, which was passed through a 395‐nm short pass filter into a 1250‐μm diameter light guide (McCarron et al., 2010; Olson, Chalmers, & McCarron, 2012). The light guide was fixed to a spot illumination adaptor, mounted onto the epi‐illuminator of the microscope and focussed using broadband light. For each imaging session, an image was taken using this broadband light to identify the uncaging region. The uncaging region had a diameter of ~70 μm.

For uncaging, second‐order mesenteric arteries were prepared as previously described and incubated with 5‐μM Cal‐520 and 5‐μM membrane‐permeant Ins(1,4,5)P3‐caged IP_3_ (SiChem) in PSS for 30 min at 37°C. Image acquisition was performed at 10 fps for 1,000 frames (1 min 40s), with uncaging performed after 15 s. Fifteen minutes were left between each uncaging event to ensure Ca^2+^ stores had fully replenished. All uncaging events were recorded in triplicate.

The endothelium was incubated with rapamycin (10 μM), FK506 (10 μM), okadaic acid (5 μM), or cypermethrin (10 μM) for 30 min before repeat recordings on the same preparation were taken. Preparations were incubated in https://www.guidetopharmacology.org/GRAC/LigandDisplayForward?ligandId=2433 (500 μM) for 10 min.

### 2.5 ACh‐stimulated Ca^2+^ signalling

Arteries were mounted in custom‐made imaging chambers. The imaging chambers were fitted with a perfusion pump (set to 1.5 ml·min^−1^) to flow solutions and an aspirator to remove waste. To avoid “run‐up” of the ACh responses (Figure S1), preparations were stimulated with ACh (50 nM) in PSS (15 min), washed with PSS (15 min) twice before the experiments were started.

Experiments were performed in triplicate, with baseline recordings followed by pharmacological perturbation. Images were acquired at 10 fps for 2,000 frames (3 min 20 s) and washed for 20 min with PSS, all performed under flow (1.5 ml·min^−1^). Following controls, arteries were incubated with FK506 (10 μM), okadaic acid (5 μM), or cypermethrin (10 μM) for 30 min under no flow conditions before repeat recordings were taken in the same preparation. Preparations were incubated in 2‐APB (500 μM) for 10 min.

### 2.6 Effects of ryanodine on Ca^2+^ signalling

Arteries were prepared as previously described and incubated with Cal‐520 (5 μM) in PSS for 30 min at 37°C. Image acquisition was performed at 10 fps for 1,000 frames (1 min 40s), with stimulation performed after 30 s. Fifteen minutes were left between each recording session. Preparations were incubated with ryanodine (30 μM), caffeine (10 mM), or combinations of both and stimulated with ACh (50 nM) as described in the text.

### 2.7 Image analysis

Image analysis was performed using methods adapted from Wilson, Lee, and McCarron (2016). In brief, cell location and shift in cell position between technical replicates were initially determined using Fiji (RRID:SCR_002285) to create matched coordinates for each cell over repeated imaging sessions. A semi‐automated Python‐based code adapted from Wilson, Lee, and McCarron (2016), Wilson, Saunter, Girkin, and McCarron (2016), and Wilson, Saunter, Girkin, and McCarron (2015) was used to extract and process Ca^2+^ signals from regions of interest placed on each cell. Raw signals were expressed as baseline‐corrected fluorescence intensity (*F*/*F*_0_) by dividing each intensity trace by an averaged 100‐frame baseline period at the start of each trace. These signals were then smoothed by a 21‐point third‐order polynomial Savitzky–Golay filter. Key curve parameters (e.g., amplitude, frequency, and number of cells) were calculated using an automated algorithm and peaks identified using a zero crossing detector for the derivative of the trace (Wilson, Lee, & McCarron, 2016). The time and amplitude of these peaks could then be extracted.

In uncaging experiments, before image analysis was performed, a mask was applied to the raw data to only analyse cells in which IP_3_ was directly uncaged. The uncaging region occupied a fraction of the overall field so these experiments had a lower number of cells per experiment than ACh‐evoked signalling.

To determine whether or not FK506 or rapamycin evoked “Ca^2+^ leak” from the internal store, the number of cells showing spontaneous Ca^2+^ activity at baseline (Frames 1–150) were manually counted for each biological replicate.

### 2.8 Endothelial patch isolation

The aorta was dissected, collected into PSS with sodium pyruvate (NaPy, 2 mM), the surrounding fat removed, washed, and pinned out, removing any remaining blood. The aorta was then cut into small strips and incubated in collagenase (Type 3, 2 mg·ml^−1^ in PSS + 2 mM NaPy) for 20 min at 37°C in a water bath. The collagenase was gently removed and PSS + 2 mM NaPy +1% BSA used to gently wash the tissue three times. A glass Pasteur pipette was cut to a length of ~2 cm, flame‐sterilized and rounded off, and used to titurate the tissue vigorously 20 times. Patches were then placed on an EtOH‐sterilized glass coverslip in an imaging chamber and left to settle for 3 hr.

### 2.9 Endothelial patch image acquisition

Patches were loaded with Cal‐520 (5 μM) for 30 min and mounted on the inverted microscope previously described. A gravity‐driven perfusion set‐up in conjunction with an aspirator was used to gently change solution without disturbing endothelial cell patches. Cells were imaged at 10 fps with PSS being gravity‐perfused to provide a baseline recording of 5000 frames (8 min 20 s). Without stopping perfusion, ACh (50 nM in PSS + 2 mM NaPy) was then administered for a further 5000 frames, to ensure endothelial cells within the patch were fully responsive. Patches were washed with PSS + 2 mM NaPy for a further 5,000 frames, and then caffeine (10 mM in PSS + 2 mM NaPy) was administered for a further 5,000 frames and the corresponding Ca^2+^ signals acquired.

### 2.10 Endothelial patch image analysis

Cells were manually identified, and the *F*/*F*_0_ intracellular Ca^2+^ levels calculated. Data were smoothed over 20 data points and the resulting traces plotted in MATLAB R2018a (The Mathworks Inc, Natick, Massachusetts, United States; RRID:SCR_001622).

### 2.11 FKBP12 immunocytochemistry

The antibody‐based procedures used in this study comply with the recommendations made by the *British Journal of Pharmacology* (Alexander et al., 2018). Second‐order mesenteric arteries were dissected and then pinned out on Sylgard bases in six well plates, fixed with freshly prepared paraformaldehyde (4%), and left overnight at 4°C. All washes were performed for 5 min unless otherwise stated. After washing three times with 0.1‐M glycine, preparations were washed three times in PBS and permeabilized for 10 min in 0.2% Triton‐X100. Preparations were then washed three times in PBS, three times in an antibody wash solution (20× SSC, 0.05% Triton‐X100 in ddH_2_0), and blocked in 5% BSA for 1 hr. Preparations were next washed three times in antibody wash solution and incubated overnight at 4°C either with the primary antibody (Abcam rabbit polyclonal Anti‐FKBP12, Ab2918) or without it (for a no‐primary control) in antibody buffer (20× SSC, 2% donkey serum, 1% BSA, 0.05% Triton X‐100, 0.02% sodium azide in ddH_2_0). Preparations were then washed three times in antibody wash solution and incubated with a secondary antibody (Invitrogen AF488 donkey anti‐rabbit, A‐21206) in antibody buffer for 1 hr at room temperature then washed three times in antibody wash solution. Immediately prior to imaging, preparations were incubated in 4‐nM DAPI for 5 min and washed with PBS. A Nikon Eclipse FNI upright microscopy body with a Nikon Fluor 40× 0.8 NA water immersion objective lens, illuminated by a pE‐4000 CoolLED system at 365 and 470 nm, was used to image the preparations; 100 images were acquired and an average image intensity projection taken using Fiji (Schindelin et al., 2012).

### 2.12 Data and statistical analysis

The data and statistical analysis comply with the recommendations of the *British Journal of Pharmacology* on experimental design and analysis in pharmacology (Curtis et al., 2018). Biological replicates are presented as graphical summary data. These were averaged, paired responses in arteries from ≥5 different animals. Mean values are shown as means ± SEM. Technical replicates are the three repeat measurements of each conditions. Raw peak *F/F*_0_ responses were log transformed to ensure normal distribution of the data, analysed statistically using either a paired Student's *t* test or a two‐way ANOVA with Tukey's multiple comparisons test on Prism, version 6.0 (GraphPad, La Jolla, CA, USA; RRID:SCR_005375), and the means back‐transformed for data presentation. *P* < .05 was considered statistically significant.

### 2.13 Materials

Ins(1,4,5)P3‐caged IP_3_ was obtained from SiChem. Cal‐520/AM, anti‐FKBP12, and donkey serum were obtained from Abcam (Cambridge, MA, USA). Pluronic F‐127 was obtained from Invitrogen (Carlsbad, CA, USA). AF488 donkey anti‐rabbit was obtained from Invitrogen. FK506, rapamycin, cypermethrin, okadaic acid, caffeine, 2‐APB, and all other chemicals were obtained from Sigma‐Aldrich (St. Louis, MO, USA). All solutions were freshly prepared each day.

### 2.14 Nomenclature of targets and ligands

Key protein targets and ligands in this article are hyperlinked to corresponding entries in http://www.guidetopharmacology.org/, the common portal for data from the IUPHAR/BPS Guide to PHARMACOLOGY (Harding *et al*., 2018), and are permanently archived in the Concise Guide to PHARMACOLOGY 2019/20 (Alexander, Fabbro *et al*., 2019a; Alexander, Mathie *et al*., 2019b).

## 3 RESULTS

### 3.1 Ca^2+^ release from the intracellular store

**Figure 1:**
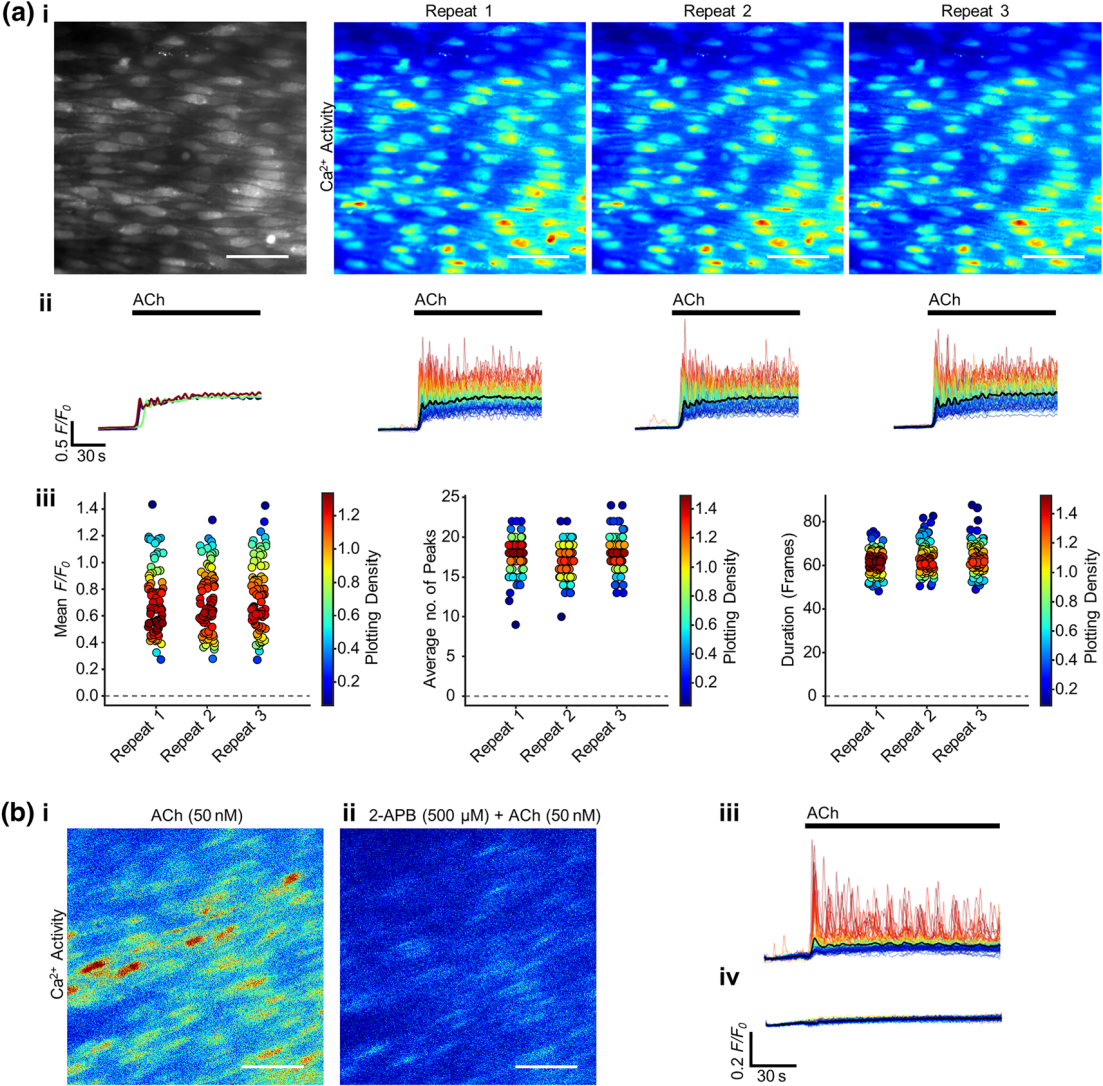
ACh evokes reproducible Ca^2+^ increases that are blocked by the IP_3_R antagonist 2‐APB. (a) Reproducible ACh‐evoked (50 nM, 1.5 ml·min^−1^) Ca^2+^ signalling in ~100 cells, (i) represented as average intensity (grayscale) and contrast‐matched images of Ca^2+^ activity (JET LUT) for each repeat ACh application (15 min apart). (ii) Individual *F/F*_0_ Ca^2+^ traces from each cell are shown, with traces coloured according to intensity of response from red to blue, and the average signal overlaid in black. The average signal for each repeat is plotted in the graph on the left. ACh activation is indicated by bars above the traces. (iii) Summary data of the mean *F/F*_0_ value from each cell, the average number of oscillation peaks per cell, and the mean duration of the responses for each of the three repeats. The colour of each point corresponds to the density of the plotted points. (b) Representative contrast‐matched (JET LUT) images of ACh‐evoked Ca^2+^ activity (i) in the absence or (ii) presence of the IP_3_R antagonist 2‐APB (500 μM, 10‐min incubation). The corresponding *F/F*_0_ traces are shown (average signal overlaid in black) (iii) in the absence or (iv) presence of 2‐APB, with the ACh application indicated with a bar above the trace. Scale bars = 50 μm

Ca^2+^ release from intracellular stores within endothelial cells may occur via IP_3_R or RyR, or both. As a first step in determining the contribution of IP_3_R and RyR, cells were activated with the IP_3_‐mobilizing agonist ACh (50 nM; Figure 1a; Video S1). ACh evoked reproducible increases in Ca^2+^ (Figure 1a(i–iii)). To verify that ACh‐evoked Ca^2+^ increases were IP_3_‐dependent, arteries were incubated with the IP_3_R antagonist 2‐APB (500 μM) for 10 min and ACh reapplied to the same preparation (Figure 1b). In the presence of 2‐APB, ACh no longer evoked Ca^2+^ increases (Figure 1b). To confirm that 2‐APB (500 μM for 10 min) was not cytotoxic, preparations were subsequently stained with propidium iodide (1.5 μM) then washed for >30mins and restimulated with ACh (Figure S2). After the protocol, cells were largely unaffected (one cell death occurred in a field of ~300 cells, yellow arrow, Figure S2B). The Ca^2+^ response to ACh was eliminated in the presence of 2‐APB but was partly restored after PSS washing (Figure S2A–D).

**Figure 2:**
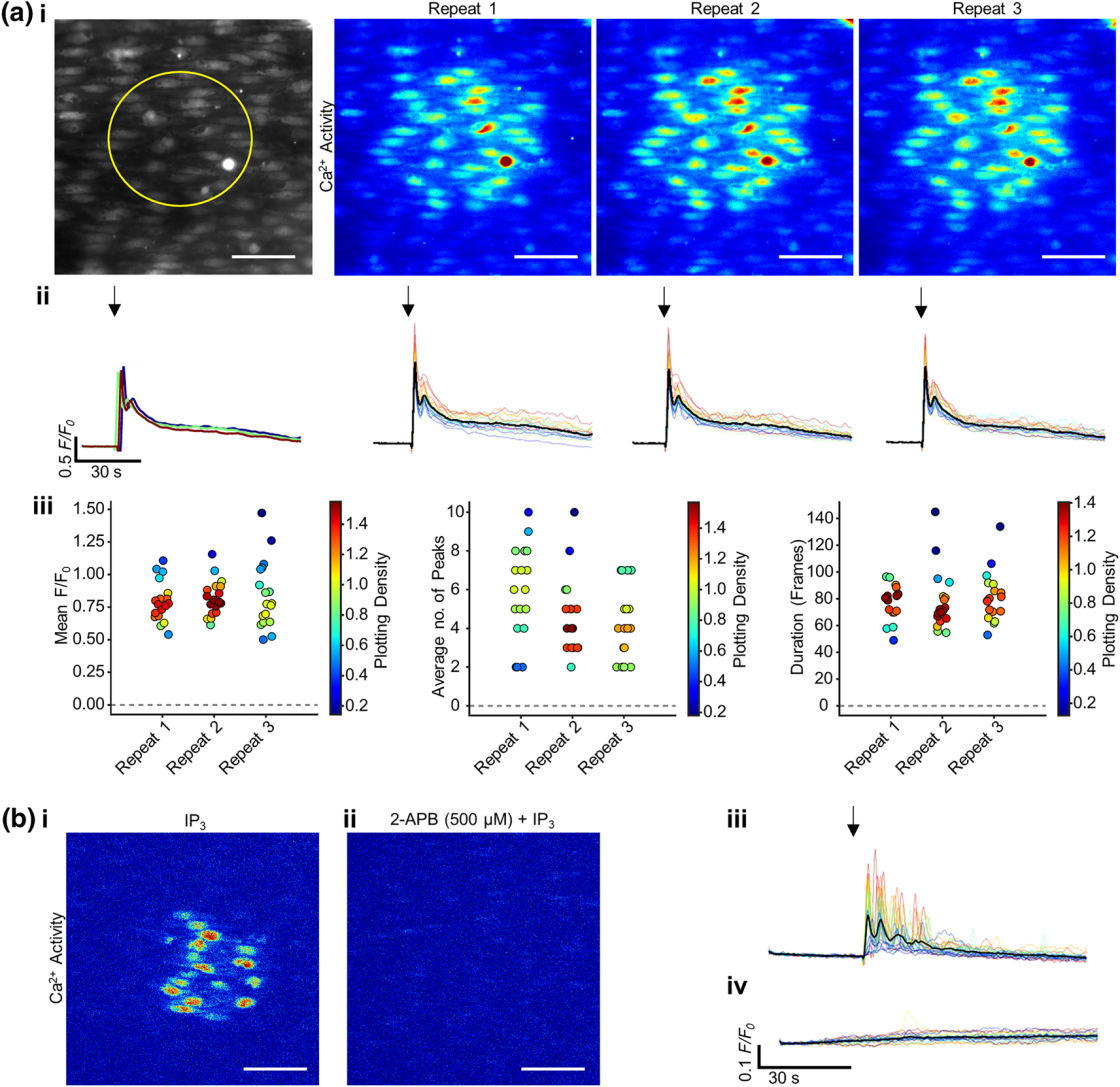
Photo‐uncaged IP_3_ evokes reproducible Ca^2+^ responses that are blocked by the IP_3_R antagonist 2‐APB. (a) Reproducible IP_3_‐activated (5 μM) Ca^2+^ signalling in ~30 endothelial cells, represented as average intensity (grayscale; left. Yellow circle represents the subsequent uncaging region) and contrast‐matched (JET LUT) images of Ca^2+^ activity each repeat application (15 min apart). (ii) Individual *F*/*F*_0_ Ca^2+^ traces from each cell are shown, with traces coloured according to intensity of response from red to blue, and the average signal overlaid in black. The average signal for each repeat is plotted in the graph on the left. Activation is indicated by an arrow above the image. Only cells in which IP_3_ was directly uncaged were analysed. (iii) Summary data on the average *F*/*F*_0_ value from each cell, the average number of oscillation peaks per cell, and the mean duration of the responses for the three repeats. The colour of each point corresponds to the density of the plotted points. (b) Representative contrast‐matched JET LUT images of IP_3_‐evoked Ca^2+^ activity (i) in the absence or (ii) presence of the IP_3_R antagonist 2‐APB (500 μM, 10‐min incubation). The corresponding *F*/*F*_*0*_ traces are shown (average signal overlaid in black) (iii) in the absence or (iv) presence of 2‐APB, with the stimulus indicated by an arrow. Scale bars = 50 μm

To confirm the efficacy of IP_3_ in evoking Ca^2+^ release, the inositide was flash‐released using a photoactivatable form of caged‐IP_3_ in a localized group of endothelial cells (Figure 2a, region highlighted by yellow circle; Video S2). Photo‐release of caged‐IP_3_ generated rapid, reproducible Ca^2+^ transients in the endothelial cells targeted for photolysis. Ca^2+^ release evoked by photolysis of caged IP_3_ was also blocked by 2‐APB (Figure 2b).

### 3.2 RyR does not contribute to Ca^2+^ signals arising from the internal store

**Figure 3:**
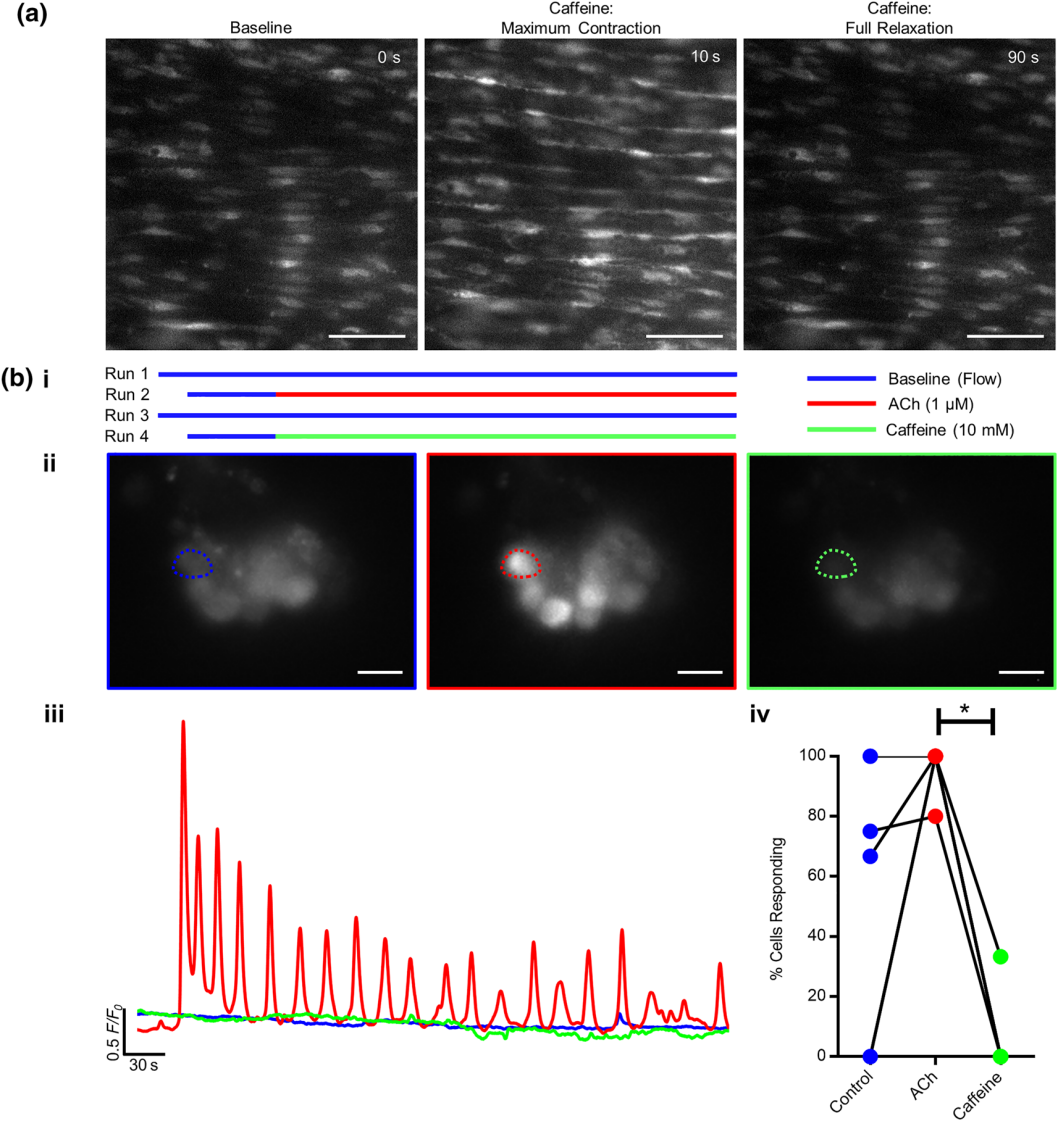
RyR do not contribute to endothelial cell Ca^2+^ release. (a) Representative images of endothelial Ca^2+^ levels in intact mesenteric arteries using the *en face* preparation. Caffeine (10 mM) evokes a substantial transient contraction of the artery. Endothelial Ca^2+^ images are shown at rest (baseline), after the maximum vessel caffeine‐evoked contraction, and after the artery returned to its initial diameter (full relaxation). All images are contrast‐level matched. The endothelial image after the maximum contraction appears brighter because of the vessel contraction squeezing the cells so that more indicator is present in the same area; see also Video S3. Scale bar = 50 μm. (b) (i) A schematic representation of the experimental protocol showing the periods of recording during each experiment. (b) (ii) Ca^2+^ images in isolated aortic endothelial cell patches in response to basal perfusion (control, left), perfusion with ACh (1 μM; middle), and after caffeine (10 mM; right; scale bar = 10 μm). (iii) *F*/*F*_0_ Ca^2+^ traces from the cells indicated in (ii). (iv) Paired summary data showing the effect of each agonist on the percentage of cells exhibiting Ca^2+^ activity. In this analysis, any activity is counted as a responding cell, and the results show that caffeine inhibits basal Ca^2+^ activity (*n* = 5, overlapping data points appear as single circles; * *P* < .05, significantly different as indicated)

To determine the extent to which RyRs contribute to Ca^2+^ release from the endothelial intracellular store, the potent RyR activator, caffeine (10 mM) was applied. While the artery rapidly responded by contracting, as the smooth muscle cells activated, there was no endothelial Ca^2+^ signal evoked by caffeine (Figure 3a; Video S3). A quantitative analysis of caffeine‐induced signal intensity was not possible because of the arterial contraction which moved cell positions, so aortic endothelial patches were dissociated from smooth muscle cells and imaged (Figure 3b). Endothelial patches were imaged first to obtain a baseline recording (8 min) in PSS flow. Next ACh (1 μM, 8 min) was applied to the endothelial patches followed by a wash period (20‐min PSS) and finally caffeine (10 mM; 8 min; Figure 3b(i–ii); Video S4). A representative image of a patch is shown in Figure 3b(ii), and the corresponding *F*/*F*_0_ traces of the highlighted cell is plotted in Figure 3b(iii). The endothelial cell showed no response to PSS flow but had a substantial Ca^2+^ response to ACh. As in the intact artery, there was no Ca^2+^ response to caffeine. On average, over five biological repeats, 96% endothelial cells analysed responded to ACh, while only 16% were active during caffeine perfusion, which was lower than the number of cells expected from baseline spontaneous activity (on average 48% cells; Figure 3b; iv). The higher percentage of cells showing basal activity is likely to be a consequence of the smaller number of cells in the endothelial patches (when compared to the intact artery), and the use of sodium pyruvate in the PSS which leads to a greater basal Ca^2+^ response to shear stress (Wilson, Lee, & McCarron, 2016). The lower number of cells showing baseline spontaneous Ca^2+^ signals in caffeine probably reflects a caffeine‐induced inhibition of IP_3_R activity that generates the spontaneous activity (Brown, Sayers, Kirk, Michell, & Michelangeli, 1992; Missiaen, Taylor, & Berridge, 1992; Parker & Ivorra, 1991; Wilson et al., 2019). These results show that ACh evokes a substantial Ca^2+^ increase while caffeine does not. Agonists of RyR do not appear to mediate Ca^2+^ release from the mesenteric or aortic endothelium.

**Figure 4:**
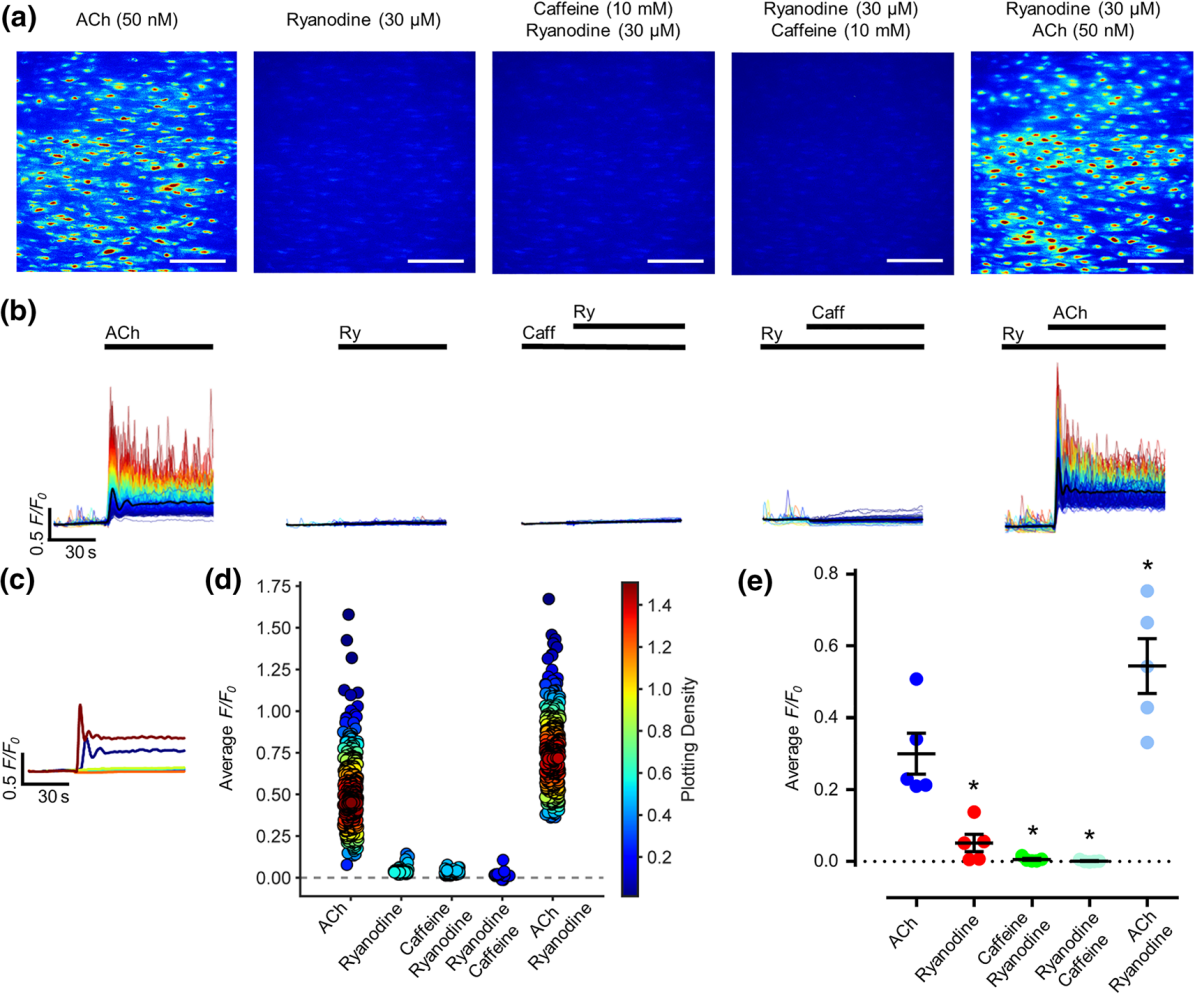
RyR does not contribute to endothelial cell Ca^2+^ release. (a) Representative, contrast‐matched images of endothelial Ca^2+^ levels in an intact mesenteric artery using the *en face* preparation after ACh (50 nM) or ryanodine (30 μM) administration, ryanodine added in the presence of caffeine (10 mM), caffeine added in the presence of ryanodine, or ACh added in the presence of ryanodine. Images are shown using JET LUT. Scale bar = 100 μm. (b) Individual *F*/*F*_0_ Ca^2+^ traces from each cell are shown for each of the above conditions. Traces are coloured according to intensity of response from red to blue, and the average signal overlaid in black. Reagents present are indicated by a bar above the image. (c) Average trace for each condition plotted alone. (d) Summary data on the average *F*/*F*_0_ value from each cell for each of the conditions. The colour of each point corresponds to the density of the plotted points. (e) Average *F*/*F*_0_ value for each biological replicate for each condition. *n* = 5; * *P* < .05, significantly different from baseline ACh recording; all data are matched

To confirm that RyRs do not mediate mesenteric endothelial Ca^2+^ regulation, after a basal ACh (50 nM) stimulation, preparations were washed and stimulated using ryanodine alone (30 μM). Preparations were washed and caffeine (10 mM) added to cause the characteristic artery contraction and relaxation. Ryanodine was then added to the caffeine‐stimulated preparations (Figure 4a–e; ). Practically no endothelial Ca^2+^ responses were elicited in response to ryanodine in the absence (0.05 ± 0.02 AFU; mean values ± SEM) or presence (0.006 ± 0.003 AFU) of caffeine, when compared with a basal ACh response (0.3 ± 0.06 AFU). Preparations were washed and again incubated with ryanodine for 15 min, followed immediately by ryanodine and caffeine stimulation (0.001 ± 0.001 AFU). Again, no response was seen in preparations. When caffeine was removed and ACh (50 nM) added to the ryanodine, a strong Ca^2+^ response was elicited (Figure 4b(i–iii); 0.54 ± 0.08 AFU). These experiments again suggest that RyR do not have a functional role in mesenteric endothelial Ca^2+^ regulation.

### 3.3 FK506 modulates IP_3_‐evoked Ca^2+^ signals by calcineurin inhibition

**Figure 5:**
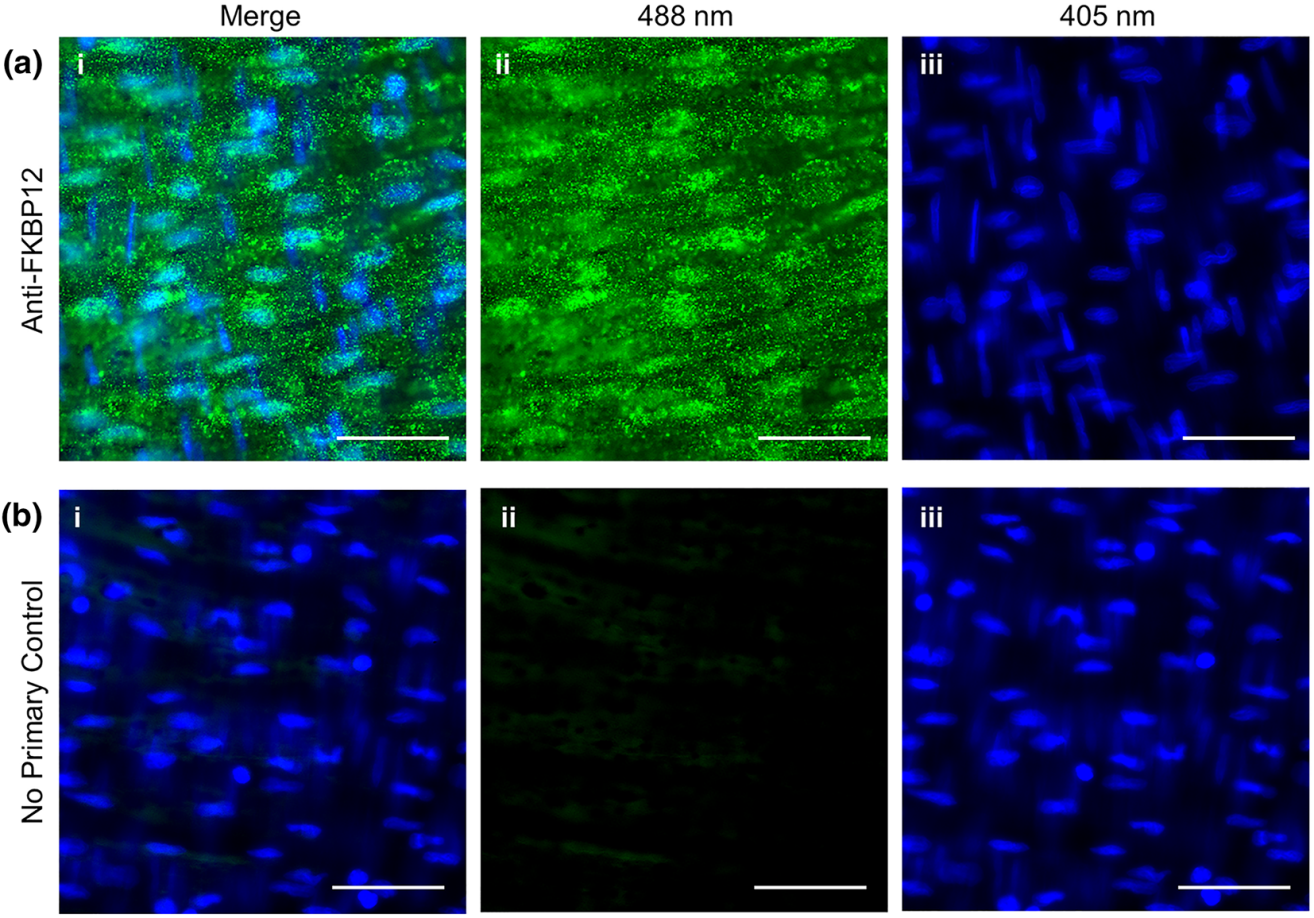
FKBP12 is present in mesenteric arterial endothelial cells. Representative *en face* mesenteric arterial preparations stained with (a) an anti‐FKBP12 antibody (green) or (b) with a no‐primary antibody control (non‐specific binding). Images are shown (i) merged, (ii) the 488‐nm channel alone, or (ii) the 405‐nm DAPI channel alone. Each image is an average projection of 100 images, and all images are contrast matched. Scale bars = 50 μm. Representative of three separate experiments

In other tissues, FKBP12 has been shown to regulate Ca^2+^ release from the internal store. However, very little is known about the role of FKBP in endothelial cell Ca^2+^ modulation. To first establish whether or not FKBP12 is present in endothelial cells, immunohistochemistry was performed (Figure 5). FKBP12 (green puncta) was present throughout the endothelial cell cytoplasm (Figure 5a(i–ii)). Staining was absent in controls that lacked the primary antibody (Figure 5b). These results confirm the presence of FKBP12 in mesenteric artery endothelial cells.

**Figure 6:**
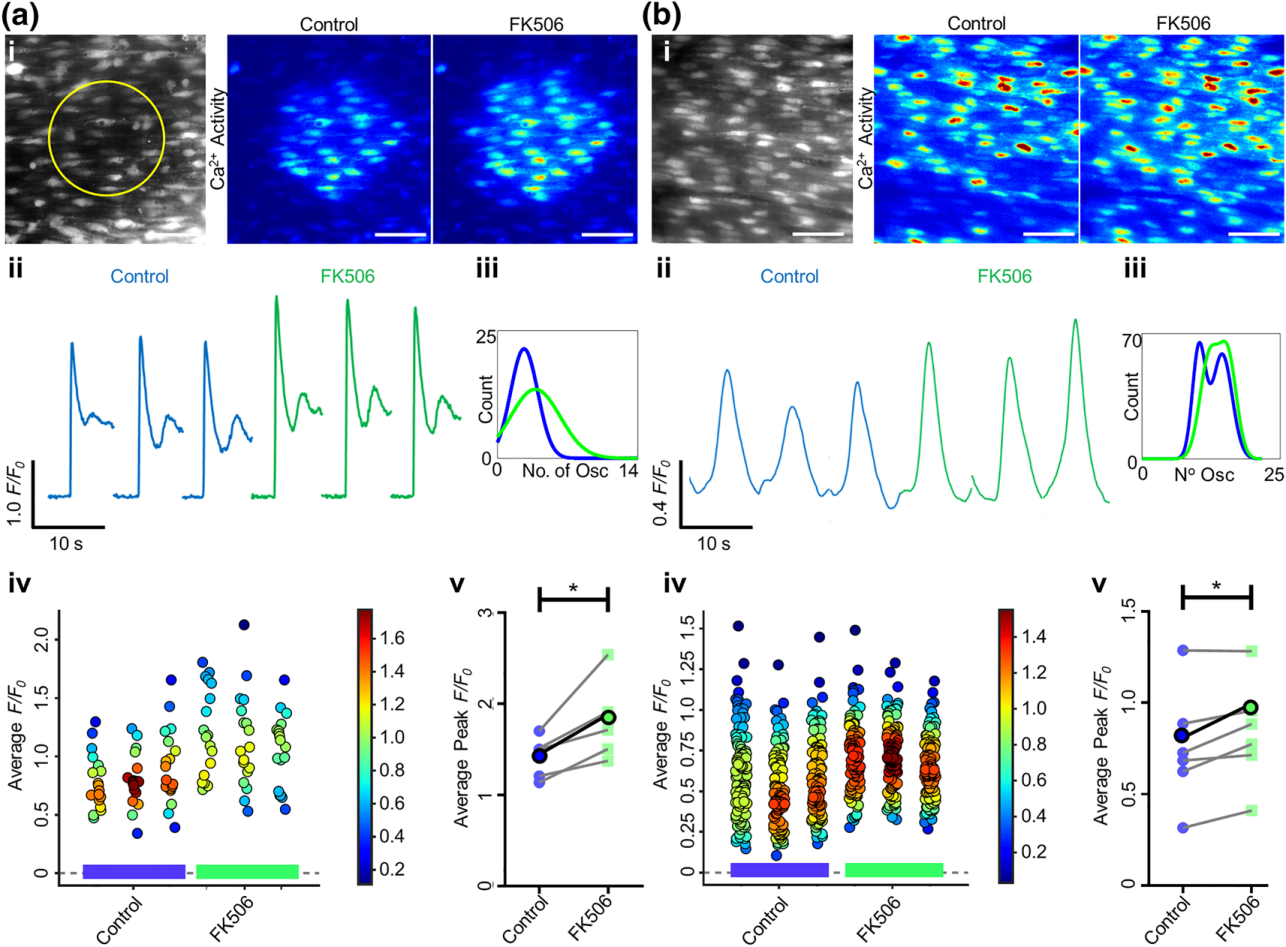
FK506 increases endothelial IP_3_‐ and ACh‐evoked Ca^2+^ release. Ca^2+^ release was stimulated via either (a) localized photolysis of caged IP_3_ (5 μM) or (b) ACh (50 nM; flow at 1.5 ml·min^−1^). Only cells within which IP_3_ was directly uncaged were analysed; therefore, more cells were analysed per artery for ACh stimulation. (i) Average intensity images of the endothelial cell field at baseline (grayscale, yellow circle represents the subsequent uncaging region) and representative contrast‐matched images (Jet LUT) of paired endothelial cell Ca^2+^ levels after IP_3_ or ACh stimulation under control conditions or after 30‐min incubation with FK506 (10 μM). Scale bars = 50 μm. (ii) Representative individual *F*/*F*_0_ traces from 10‐s recordings after stimulation for a single cell are shown. Control (blue) and FK506‐treated (green) traces are compared for each technical replicate within an experiment. (iii) Fitted curves for histograms illustrating the spread of the average number of oscillations per cell for control and FK506‐incubated conditions across all technical and biological repeats. (iv) Summary data for the average *F*/*F*_0_ value from each cell (colour corresponds to the density of the plotted points). Data are technical repeats from a single experiment, with control and FK506‐incubated recordings indicated with a blue or green bar beneath the data respectively. (v) Peak *F*/*F*_0_ signals averaged for each cell and across three technical replicates for each biological repeat, compared between control and FK506. Each biological replicate is shown, with the average highlighted in bold. *n* = 5,6; * *P* < .05, significantly different as indicated

FK506 disrupts binding of FKBP to IP_3_R and may result in “leaky” channels (Liu et al., 2018) as occurs with RyR (Long et al., 2007). However, the present results showed no change in basal Ca^2+^ signalling activity in the presence of FK506 (Figure S3) suggesting no change in channel “leak.” To further investigate the role FKBP12 plays in endothelial Ca^2+^ regulation, IP_3_Rs were stimulated before and after incubation with FK506 (10 μM). In each experiment, ~100 endothelial cells were studied, and all comparisons were matched between cells in the same preparation. IP_3_R activation was achieved either by flash release of a caged form of IP_3_ (5 μM) in a localized group of endothelial cells (Figure 6a) or by ACh (50 nM; 1.5 ml·min^−1^, Figure 6b).

FK506 evoked an increase in the Ca^2+^ transient generated by each method of IP_3_R activation (Figure 6i,ii). The peak *ΔF*/*F*_0_ amplitude was significantly increased after FK506 incubation for IP_3_‐evoked (*n* = 5, 1.41 ± 0.10 to 1.81 ± 0.20) and ACh‐evoked (*n* = 6, 0.78 ± 0.1 to 0.85 ± 0.1) signals (Figure 6v). Likewise, the average cell Δ*F*/*F*_0_ (Figure 6iv) increased after FK506 incubation for both IP_3_ and ACh stimulation. Analysis of the distribution of Ca^2+^ oscillations shows a shift towards a larger number of oscillations after incubation with FK506 under both types of IP_3_R stimulation (Figure 6iii).

**Figure 7:**
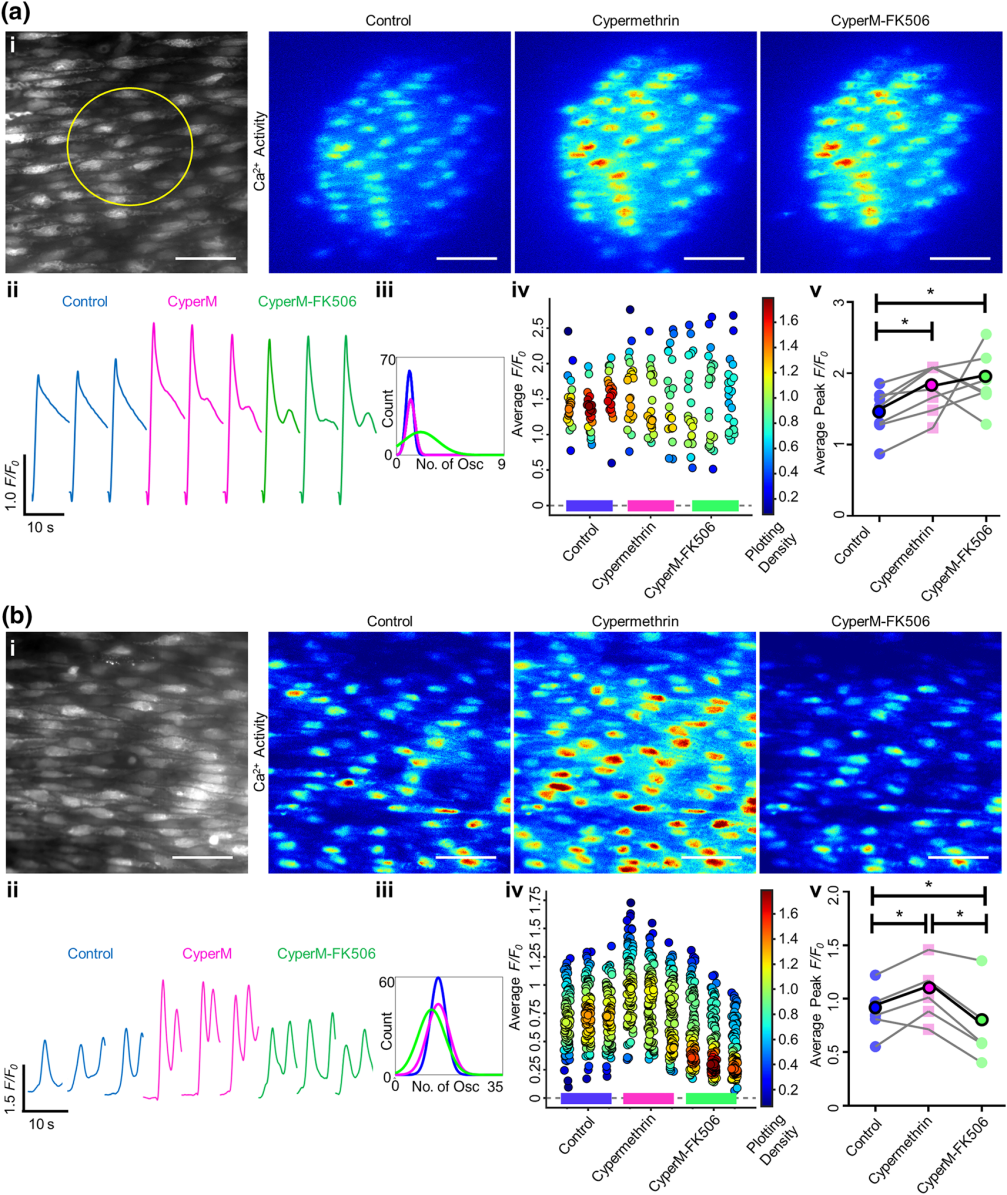
Cypermethrin increases Ca^2+^ release and blocks FK506‐induced increase in IP_3_‐ and ACh‐evoked Ca^2+^ release. Ca^2+^ release was stimulated by either localized photolysis of caged IP_3_ (5 μM) (a) or (b) ACh (50 nM; flow at 1.5 ml·min^−1^). Only cells within which IP_3_ was directly uncaged were analysed; therefore, more cells were analysed per artery for ACh stimulation. (i) Average intensity images of the endothelial cell field at baseline (grayscale, yellow circle represents the subsequent uncaging region) and representative contrast‐matched images (Jet LUT) of paired endothelial cell Ca^2+^ levels after IP_3_ or ACh stimulation under control, cypermethrin (CyperM; 10 μM, 30‐min incubation), or cypermethrin‐FK506 (10 μM of each drug, 30‐min incubation) conditions. Scale bars = 50 μm. (ii) Representative individual *F*/*F*_*0*_ traces from 10‐s recordings after stimulation for a single cell are shown. Control, cypermethrin and both cypermethrin‐ and FK506‐treated traces are compared for each technical replicate within an experiment. (iii) Fitted curves for histograms illustrating the spread of the average number of oscillations per cell for control, cypermethrin‐incubated and cypermethrin‐FK506‐incubated conditions, across all technical and biological repeats. (iv) Summary data for the average *F*/*F*_0_ value from each cell (colour corresponds to the density of the plotted points). Data are technical repeats from a single experiment, with control, cypermethrin‐incubated, and cypermethrin‐ and FK506‐incubated recordings indicated with a blue, pink, or green bar beneath the data respectively. (v) Peak *F*/*F*_0_ signals averaged for each cell and across three technical replicates for each biological repeat, compared between control, cypermethrin and both FK506 and cypermethrin incubation. Each biological replicate is shown, with the average highlighted in bold. (*n* = 5 for IP_3_, *n* = 7 for ACh; *P < .05, significantly different as indicated)

**Figure 8:**
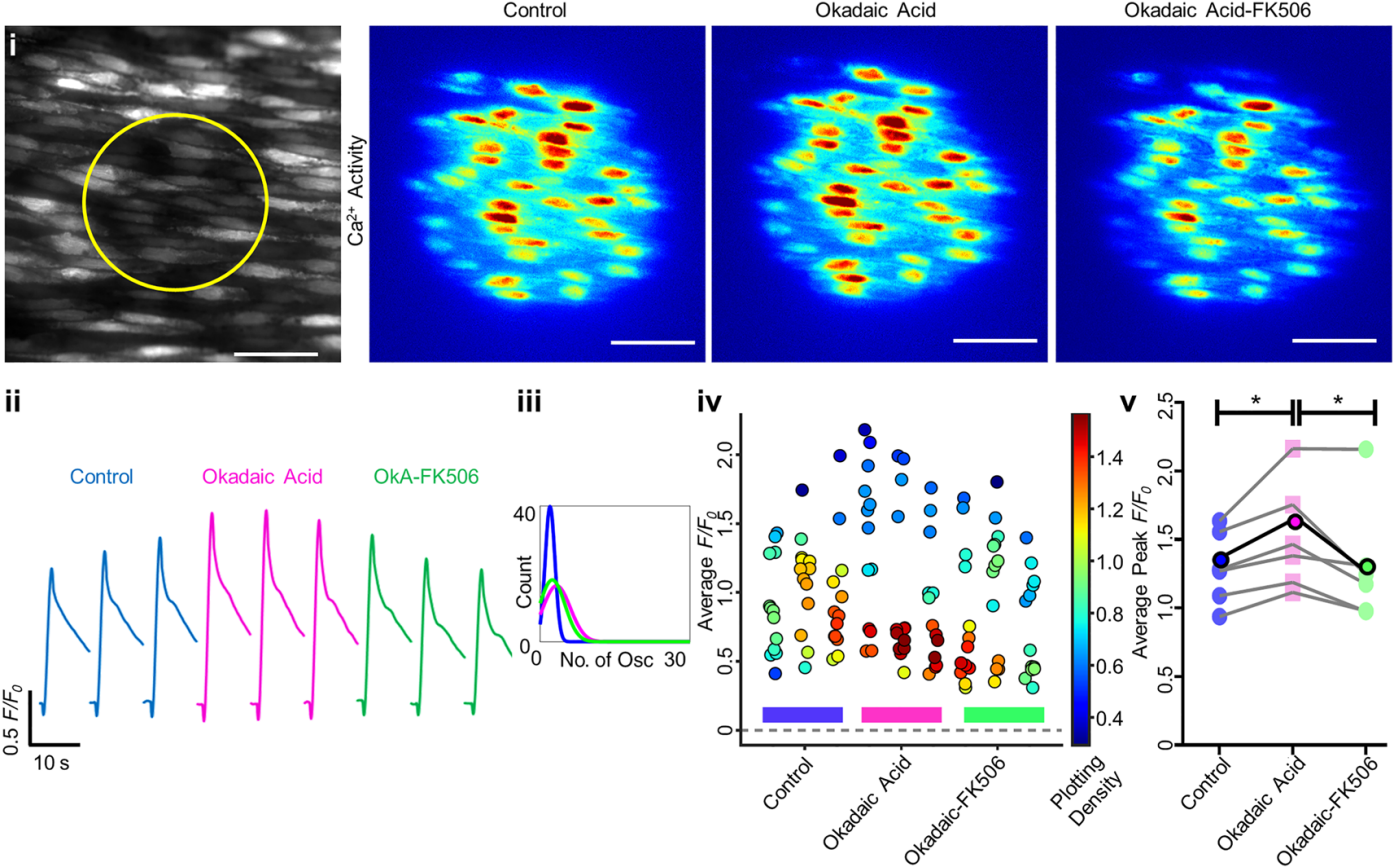
Okadaic acid increases Ca^2+^ release and blocks FK506‐induced increase in endothelial IP_3_‐ and ACh‐evoked Ca^2+^ release. Ca^2+^ release was stimulated via localized photolysis of caged IP_3_ (5 μM). (i) Average intensity images of paired endothelial cell field at baseline (grayscale, yellow circle represents the subsequent uncaging region) and representative contrast‐matched images (Jet LUT) of the endothelial cell Ca^2+^ levels after IP_3_ stimulation under control, okadaic acid (OkA; 10 μM, 30 min incubation), or okadaic acid‐FK506 (10 μM each drug, 30‐min incubation) conditions. Scale bar = 50 μm. (ii) Representative individual *F*/*F*_0_ traces from 10‐s recordings after stimulation for a single cell are shown. Control, okadaic acid and both okadaic acid‐ and FK506‐treated traces are compared for each technical replicate within an experiment. (iii) Fitted curves for histograms illustrating the spread of the average number of oscillations per cell for control, okadaic acid and okadaic acid‐FK506, across all technical and biological repeats. (iv) Summary data for the average *F*/*F*_0_ value from each cell (colour corresponds to the density of the plotted points). Data are technical replicates from a single experiment, with control, okadaic acid‐incubated, and okadaic acid‐ and FK506‐incubated recordings indicated with a blue, pink, or green bar beneath the data respectively. (v) Peak *F*/*F*_0_ signals averaged for each cell and across three technical replicates for each biological repeat, compared between control, okadaic acid and both FK506 and okadaic acid conditions. Each biological replicate is shown, with the average highlighted in bold. *n* = 5, *; *P* < .05, significantly different as indicated

The FKBP‐FK506 complex inhibits calcineurin. To determine whether or not the FK506‐induced increase in Ca^2+^ store release was due to inhibition of calcineurin, the inhibitors cypermethrin (10 μM) or okadaic acid (5 μM) were each used. Ca^2+^ release (peak ΔF/F_0_) was significantly increased after incubation (30 min) with either cypermethrin (Figure 7v, *n* = 7, 1.45 ± 0.14 to 1.73 ± 0.14, for IP_3_ activation in panel a, *n* = 5, 0.88 ± 0.11 to 1.05 ± 0.12, for ACh activation in panel b) or okadaic acid (Figure 8v, *n* = 6, 1.30 ± 0.12 to 1.52 ± 0.17). The increase was also evident in the Ca^2+^ signal images (Figures 7a(i), 7b(i), and 8i), the individual *F*/*F*_0_ traces (Figures 7a(ii), 7b(ii), and 8ii), and the average cell Δ*F*/*F*_0_ values (Figures 7a(iv), 7b(iv), and 8iv). These results suggest that the phosphatase calcineurin limits IP_3_R activity.

Significantly, in the presence of cypermethrin, FK506 failed to increase IP_3_‐mediated Ca^2+^ release (Figure 7a(v), *n* = 7, 1.73 ± 0.14 to 1.90 ± 0.19). After cypermethrin, FK506 also failed to increase ACh‐evoked Ca^2+^ release (Figure 7b(v), *n* = 5, 1.05 ± 0.12 to 0.75 ± 0.16). After the phosphatase inhibitor okadaic acid, FK506 significantly *reduced* IP_3_‐mediated Ca^2+^ release (Figure 8v, *n* = 6, 1.52 ± 0.17 to 1.33 ± 0.19). There was no significant difference in the number of ACh‐activated Ca^2+^ oscillations after incubation with FK506 and cypermethrin or okadaic acid (Figures 7a(iii), 7b(iii), and 8iii). These results suggest that the increase in IP_3_‐evoked Ca^2+^ release generated by FK506 is mediated by an effect of the drug on calcineurin rather than FKBP modulation itself.

### 3.4 Rapamycin does not alter IP_3_‐evoked Ca^2+^ signals

**Figure 9:**
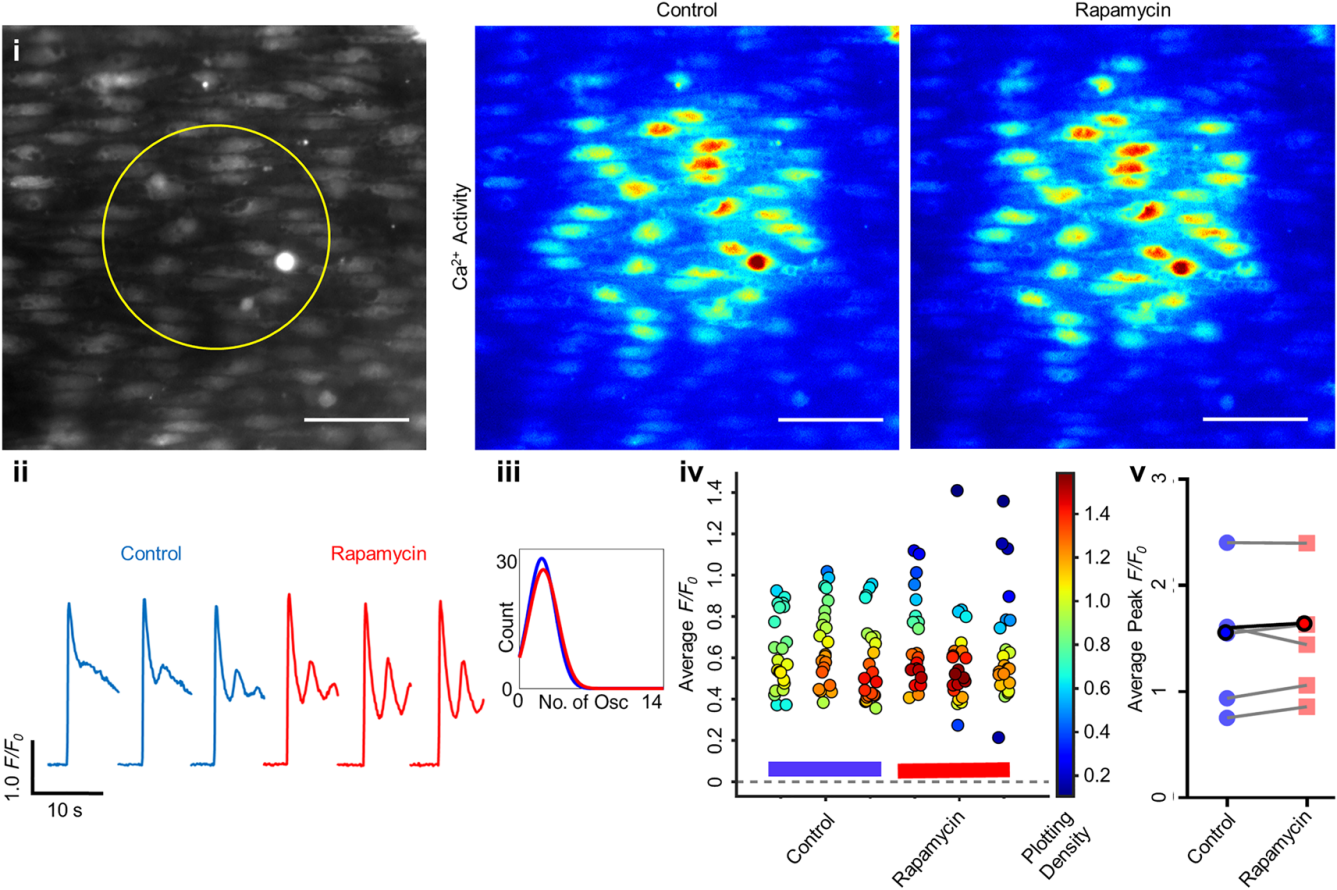
Rapamycin does not alter endothelial IP_3_‐evoked Ca^2+^ release. Ca^2+^ release was stimulated via localized photolysis of caged IP_3_ (5 μM) under no flow conditions. (i) Average intensity images of paired endothelial cell field at baseline (grayscale, yellow circle represents the subsequent uncaging region) and representative contrast‐matched images (Jet LUT) of the endothelial cell Ca^2+^ levels after IP_3_ stimulation under control or rapamycin (10 μM, 30 min incubation) conditions. Scale bar = 50 μm. (ii) Representative individual *F*/*F*_0_ traces from 10‐s recordings after stimulation for a single cell are shown. Control (blue) and rapamycin (red) traces are compared for each technical replicate within an experiment. (iii) Fitted curves for histograms illustrating the spread of the average number of oscillations per cell for control and rapamycin conditions across all technical and biological repeats. (iv) Summary data for the average *F*/*F*_0_ value from each cell (colour corresponds to the density of the plotted points). Data are technical replicates from a single experiment, with control and rapamycin recordings indicated with a blue or red bar beneath the data respectively. (v) Peak *F*/*F*_0_ signals averaged for each cell and across three technical replicates for each biological repeat, compared between control and rapamycin conditions. Each biological replicate is shown, with the average highlighted in bold. *n* = 5

Rapamycin also disrupts FKBP12 binding to IP_3_R but, unlike FK506, does not inhibit calcineurin. Rapamycin did not alter IP_3_‐evoked Ca^2+^ release. Ca^2+^ release was stimulated by localized photolysis of caged IP_3_ (Figure 9). There was no difference in the peak amplitude of IP_3_‐evoked *F*/*F*_0_ amplitude (Figure 9i–v, *n* = 5, 1.45 ± 0.25 to 1.48 ± 0.23), or in the characteristics of the subsequent Ca^2+^ oscillatory profile (Figure 9iii) after rapamycin (10 μM, 30 min). Together, these results suggest FKBP is not directly involved in endothelial IP_3_R regulation.

## 4 DISCUSSION

The immunosuppressant drugs FK506 and rapamycin are routinely used after organ transplantation and are effective at lowering organ rejection, though a major side effect of the treatment is hypertension (Chiasson et al., 2011; De Lima et al., 1999; Reis et al., 2009). Increased vascular resistance (Spencer, Goa, & Gillis, 1997; Textor et al., 1993) as a result of altered endothelial function and decreased production of the vasodilator NO appears central to the development of hypertension (De Lima et al., 1999; Takeda et al., 1999). However, while immunosuppressant‐induced hypertension coupled with endothelial dysfunction is significant in the systemic circulation, this may not be the case in the pulmonary circulation. Rather than being induced, pulmonary hypertension is suppressed by rapamycin (Houssaini et al., 2013) as a result of decreased smooth muscle proliferation. This finding points to significant differences in the pulmonary and systemic circulations and that the antiproliferative effects of rapamycin are insufficient to offset other effects of the drug in generating systemic hypertension.

The cellular mechanisms that give rise to decreased NO production and endothelial dysfunction evoked by FK506 and rapamycin are not fully understood though alterations in normal Ca^2+^ handling may be key. In other cell types, FK506 and rapamycin exert multiple effects on cellular function by modulating Ca^2+^ signalling. FK506 and rapamycin alter binding of FKBP to RyR and IP_3_R which may regulate channel activity. FK506‐FKBP additionally inhibits the phosphatase calcineurin. Here, in native endothelial cells on intact blood vessels, we explored the possibility that FKBP binding to IP_3_R and RyR may regulate Ca^2+^ signalling and thus contribute to the effects of the immunosuppressants on vascular function.

Our results suggest endothelial IP_3_R activity is not directly modulated by FKBP. In support, FKBP modulation with rapamycin did not alter IP_3_‐evoked Ca^2+^ release. On the other hand, FK506 did increase IP_3_‐evoked Ca^2+^ release, but that modulation was likely to be indirect and exerted via inhibition of calcineurin. Okadaic acid and cypermethrin, known inhibitors of calcineurin, also increased IP_3_‐evoked Ca^2+^ release. Significantly, calcineurin inhibitors blocked FK506‐evoked Ca^2+^ increases, and FK506 *decreased* IP_3_‐evoked Ca^2+^ release in the presence of okadaic acid. This finding raises the possibility that FK506 by itself may inhibit IP_3_R in the endothelium, an effect that is normally offset by potentiation of Ca^2+^ release, mediated by inhibition of calcineurin. The precise mechanisms by which calcineurin inhibition potentiates IP_3_‐evoked Ca^2+^ release is not clear but presumably arises from a direct or indirect influence of a dephosphorylation event on IP_3_R. Interestingly, caged IP_3_ and ACh elicited a different response to dual cypermethrin‐FK506 incubation, though the reasons behind this remain unclear. Nor are FK506 and rapamycin likely to mediate their effects on the endothelium via RyR. The RyR activator caffeine failed to evoke Ca^2+^ changes in the endothelium, and ryanodine itself did not alter Ca^2+^ release in pre‐activated preparations, suggesting that RyR is not functional in the native endothelium. Together these results suggest that FKBPs do not regulate Ca^2+^ release via IP_3_R or RyR in endothelial cells and that the hypertensive effects of FK506 and rapamycin may be exerted at sites separate from the documented effects of these two drugs on FKBP‐mediated Ca^2+^ release from IP_3_R or RyR.

While the present results suggest that FKBP modulation by FK506 and rapamycin do not alter Ca^2+^ release, several studies show changes in endothelial function evoked by the immunosuppressants. FK506 results in endothelial tube malformation in cell cultures (Eguchi et al., 2013; Wilasrusmee et al., 2003) and a reduced endothelium‐dependent relaxation in mouse aorta (Chiasson et al., 2011; Cook et al., 2009 ; Long et al., 2007). As relaxation in response to the smooth muscle activator sodium nitroprusside was reported to be normal (Chiasson et al., 2011), this suggests that FK506 acted via the endothelium rather than smooth muscle. Furthermore, FK506 and rapamycin each decreased NO production in mouse and rat aorta (Long et al., 2007; Takeda et al., 1999). In rat mesenteric artery, FK506 also increased the production of the endothelium‐derived smooth muscle contractile agent endothelin (Takeda et al., 1999). These studies suggest that the immunosuppressants evoke changes in endothelial function, which could underlie the development of vascular dysfunction and hypertension.

However, in other studies, more widespread effects of the drugs have been reported. In human and rat resistance arteries, in vitro exposure to FK506 for 24hr increased sensitivity to noradrenaline and impaired endothelium‐dependent relaxation to ACh (De Lima et al., 1999). However, endothelium‐independent relaxation to sodium nitroprusside was also reduced (De Lima et al., 1999), suggesting that both endothelial and smooth muscle function may be affected by FK506. In the same study (De Lima et al., 1999), rats given FK506 chronically for 8 days showed a reduced artery sensitivity to noradrenaline and a normal endothelium‐dependent relaxation (De Lima et al., 1999). These results highlight time‐dependent and complex changes in vascular responses evoked by this immunosuppressant. FK506 itself was found to evoke smooth muscle contraction in rat renal arteries (Schwertfeger et al., 2001) and to increase the sensitivity to contractile activation (noradrenaline) in rat mesenteric and human subcutaneous arteries (De Lima et al., 1999; Schwertfeger et al., 2001). However, the drug did not to induce contraction in bovine renal, mesenteric, coronary, or carotid arteries (Epstein et al., 1998) and inhibited contraction induced by high K^+^ depolarization and U46619 in porcine coronary artery (Yasutsune et al., 1999). Collectively, these studies create ambiguity about the vascular site on which the drugs work—the endothelium, smooth muscle, both, or neither—to produce hypertension.

The heart of the problem is that it is unclear how FKBP‐RyR and FKBP‐IP_3_R interactions regulate Ca^2+^ signalling in endothelial beds across the body. In cultured mouse aortic endothelial cells, depletion of FKBP increased intracellular Ca^2+^ leak via RyR (Cook et al., 2009; Long et al., 2007). In human cultured aortic endothelial cells, rapamycin increased intracellular Ca^2+^ which was prevented by ryanodine (Habib et al., 2013). However, no change in basal Ca^2+^ activity, therefore no Ca^2+^ leak, occurred after FK506 or rapamycin in the present study. Neither could we detect any caffeine‐ or ryanodine‐evoked RyR‐mediated Ca^2+^ signals, despite caffeine effectively evoking smooth muscle contraction in the same tissue. These results suggest that RyR may not be functional in native aortic or mesenteric endothelium in intact blood vessels. The reason for the difference between our results and previous findings (Cook et al., 2009; Long et al., 2007) is unclear, though it may lie in the changes undergone by endothelial cells during culture (Cook et al., 2009; Long et al., 2007). Alternatively, differences of endothelial reactivity to activators of RyR and FK506 and rapamycin may exist across species, blood vessel type, diseased state, age, and duration of exposure to the immunosuppressants. These differences could lead to different results in Ca^2+^ dynamics in vascular endothelial cells.

There are no previous reports on the effects of FK506 or rapamycin on IP_3_‐evoked Ca^2+^ release in endothelial cells. The present findings show that FK506 increased IP_3_‐evoked Ca^2+^ release via inhibition of calcineurin and that modulation of FKBP did not alter IP_3_‐evoked Ca^2+^ release, as shown using rapamycin. To ensure the effects of FK506 were mediated via IP_3_R activity rather than the generation of IP_3_ or surface receptors generating IP_3_, we directly activated IP_3_R using photolysis of caged IP_3_. The FK506‐evoked increase in IP_3_‐mediated Ca^2+^ release was prevented when the phosphatase calcineurin was blocked, which *inhibited* and in the case of okadaic acid, actually *decreased* further FK506‐mediated Ca^2+^ release. These findings support the suggestion that FK506 may inhibit IP_3_R in endothelial cells by a mechanism that is independent of either FKBP or calcineurin.

Calcineurin appears to regulate IP_3_‐evoked Ca^2+^ release in the endothelium, potentially by forming part of the FKBP‐IP_3_R channel complex (Bandyopadhyay, Shin, Ahn, & Kim, 2000; Cameron et al., 1997; Cameron, Steiner, Roskams, et al., 1995; Cameron, Steiner, Sabatini, et al., 1995; Shin et al., 2002) as FK506‐FKBP inhibits the phosphatase calcineurin. However, there is also a complex literature on the contribution of calcineurin to the control of IP_3_‐evoked Ca^2+^ release. Calcineurin inhibitors increased ATP‐induced Ca^2+^ release and IP_3_R activity in COS‐7 cells and cerebellar microsomes respectively (Bandyopadhyay, Shin, & Kim, 2000; Cameron, Steiner, Roskams, et al., 1995), and expression of a constitutively active form of calcineurin inhibited Ca^2+^ release (Bandyopadhyay, Shin, & Kim, 2000). On the other hand, the calcineurin inhibitors cypermethrin and okadaic acid did not alter Ca^2+^ release via either RyR (MacMillan et al., 2008) or IP_3_R (Macmillan & McCarron, 2009) in aortic myocytes. Neither did okadaic acid alter Ca^2+^ in porcine coronary artery (Ashizawa et al., 1989; Hirano et al., 1989). Cyclosporin A, a calcineurin inhibitor, did not alter Ca^2+^ release in pulmonary (Zheng et al., 2004) and coronary artery myocytes (Frapier et al., 2001) or SR [Ca^2+^] in aortic myocytes (Avdonin et al., 1999). These results suggest that there is also a complex relationship between calcineurin and regulation of Ca^2+^ release in the endothelium presumably as a result of the nature of the kinases that are active in cells, the phosphorylation/dephosphorylation status of IP_3_R, and the balance of activation and inhibition of the IP_3_ receptor.

In summary, the mechanisms underlying the immunosuppressant‐mediated hypertension and endothelial dysfunction in organ transplant patients remain unclear. Hypertension induced by genetic deletion of FKBP12.6 in mice is consistent with a role for FKBPs in the process (Long et al., 2007). Previous studies suggest that FK506 and rapamycin may exert vascular effects through altered Ca^2+^ signalling mediated by displacement of FKBP from IP_3_R or through FK506‐induced inhibition of calcineurin. The present findings show that rapamycin had no effect on IP_3_‐mediated Ca^2+^ release and that the increase in FK506‐mediated Ca^2+^ release was due to FK506‐FKBP calcineurin inhibition. Calcineurin inhibition also prevented FK506‐mediated IP_3_‐evoked Ca^2+^ release. While FK506 and rapamycin share the common side effect of evoking hypertension in patients, these immunosuppressants do not share a common effect on endothelial IP_3_‐evoked Ca^2+^ release. This raises the possibility that modulation of Ca^2+^ release may not be the only factor contributing to immunosuppressant drug‐induced hypertension. FK506 is also known to induce endothelial dysfunction through attenuation of https://www.guidetopharmacology.org/GRAC/FamilyDisplayForward?familyId=285 and https://www.guidetopharmacology.org/GRAC/FamilyDisplayForward?familyId=514 independently of calcineurin inhibition (Eguchi et al., 2013). Other FKBPs may also be the target of the drugs. FKBP51 and FKBP52 regulate steroid receptor signalling and store operated Ca^2+^ entry and some TRP channels (Hamilton et al., 2018; Kadeba et al., 2013; Sinkins, Goel, Estacion, & Schilling, 2004) to modulate cell function. The present findings suggest that rapamycin and FKBP do not modulate Ca^2+^ release. However, IP_3_‐mediated Ca^2+^ release may be modulated (a) by FK506 via the phosphatase calcineurin to increase Ca^2+^ release, and (b) by FK506 directly inhibiting IP_3_R.

## DECLARATION OF TRANSPARENCY AND SCIENTIFIC RIGOUR

This Declaration acknowledges that this paper adheres to the principles for transparent reporting and scientific rigour of preclinical research as stated in the BJP guidelines for https://doi.org/10.1111/bph.14207, https://doi.org/10.1111/bph.14208, and https://doi.org/10.1111/bph.14206, and as recommended by funding agencies, publishers and other organisations engaged with supporting research..

## CONFLICT OF INTEREST

The authors declare no conflicts of interest.

## AUTHOR CONTRIBUTIONS

C.B., C.W., and J.G.M. developed the concept. C.B. performed the experiments. C.B. and J.G.M. drafted the manuscript. C.B. C.W., and J.G.M. edited the manuscript. C.W. and J.G.M. sourced funding. All authors approved the final version of the manuscript.

## Supporting information

Data S1. Supporting information

Figure S1. Repeated ACh administration to mesenteric endothelial cells leads to an increase in signal (‘run‐up’) unless appropriate washes are performed
Figure S2. Endothelial function is partially recoverable after 2‐APB
Figure S3. FK506 or rapamycin did not cause Ca^2+^ leak

Video S1. Supporting information

Video S2. Supporting information

Video S3. Supporting information

Video S4. Supporting information
