# Supplementary material for: FK506 regulates Ca2+ release evoked by inositol 1,4,5‐trisphosphate independently of FK‐binding protein in endothelial cells
Source: Br J Pharmacol. 2020 Jan 26;177(5):1131–49. doi: 10.1111/bph.14905 (PMC7042112; doi:10.1111/bph.14905)
Supplement: Supplementary file 1 — Data S1. Supporting information [file BPH-177-1131-s006.docx]

**Supporting Information**

**List of IUPHAR Hyperlinks**

FK506 Regulates IP_3_-evoked Ca^2+^ release independently of FKBP in Endothelial Cells

Charlotte Buckley, Calum Wilson & John G. McCarron*

Strathclyde Institute of Pharmacy and Biomedical Science, University of Strathclyde,

161 Cathedral Street, Glasgow, G4 0RE, UK

* To whom correspondence should be addressed: John G McCarron, Strathclyde Institute of Pharmacy and Biomedical Science, 161 Cathedral Street, Glasgow, G4 0RE; john.mccarron@strath.ac.uk; Tel +44 (0)141 548 4119

Ryanodine: <https://www.guidetopharmacology.org/GRAC/LigandDisplayForward?ligandId=4303>

RyRs: <https://www.guidetopharmacology.org/GRAC/FamilyDisplayForward?familyId=125>

IP3: <https://www.guidetopharmacology.org/GRAC/LigandDisplayForward?ligandId=4222>

IP_3_Rs: <https://www.guidetopharmacology.org/GRAC/FamilyDisplayForward?familyId=123>

Caffeine: <https://www.guidetopharmacology.org/GRAC/LigandDisplayForward?ligandId=407>

ACh: <https://www.guidetopharmacology.org/GRAC/LigandDisplayForward?ligandId=294>

2-APB: <https://www.guidetopharmacology.org/GRAC/LigandDisplayForward?ligandId=2433>

FK506: <https://www.guidetopharmacology.org/GRAC/LigandDisplayForward?ligandId=6784>

Rapamycin: <https://www.guidetopharmacology.org/GRAC/LigandDisplayForward?ligandId=6031>

FKBP: <https://www.guidetopharmacology.org/GRAC/ObjectDisplayForward?objectId=2609>

Okadaic Acid: <https://www.guidetopharmacology.org/GRAC/LigandDisplayForward?ligandId=5349>
