# Supplementary material for: FK506 regulates Ca2+ release evoked by inositol 1,4,5‐trisphosphate independently of FK‐binding protein in endothelial cells
Source: Br J Pharmacol. 2020 Jan 26;177(5):1131–49. doi: 10.1111/bph.14905 (PMC7042112; doi:10.1111/bph.14905)
Supplement: Supplementary file 2 — Figure S1. Repeated ACh administration to mesenteric endothelial cells leads to an increase in signal (‘run‐up’) unless appropriate washes are performed Figure S2. Endothelial function is partially recoverable after 2‐APB Figure S3. FK506 or rapamycin did not cause Ca2+ leak [file BPH-177-1131-s001.docx]

**SUPPLEMENTARY INFORMATION**

FK506 Regulates IP_3_-evoked Ca^2+^ release independently of FKBP in Endothelial Cells

Charlotte Buckley, Calum Wilson & John G. McCarron*

Strathclyde Institute of Pharmacy and Biomedical Science, University of Strathclyde,

161 Cathedral Street, Glasgow, G4 0RE, UK

* To whom correspondence should be addressed: John G McCarron, Strathclyde Institute of Pharmacy and Biomedical Science, 161 Cathedral Street, Glasgow, G4 0RE; john.mccarron@strath.ac.uk; Tel +44 (0)141 548 4119


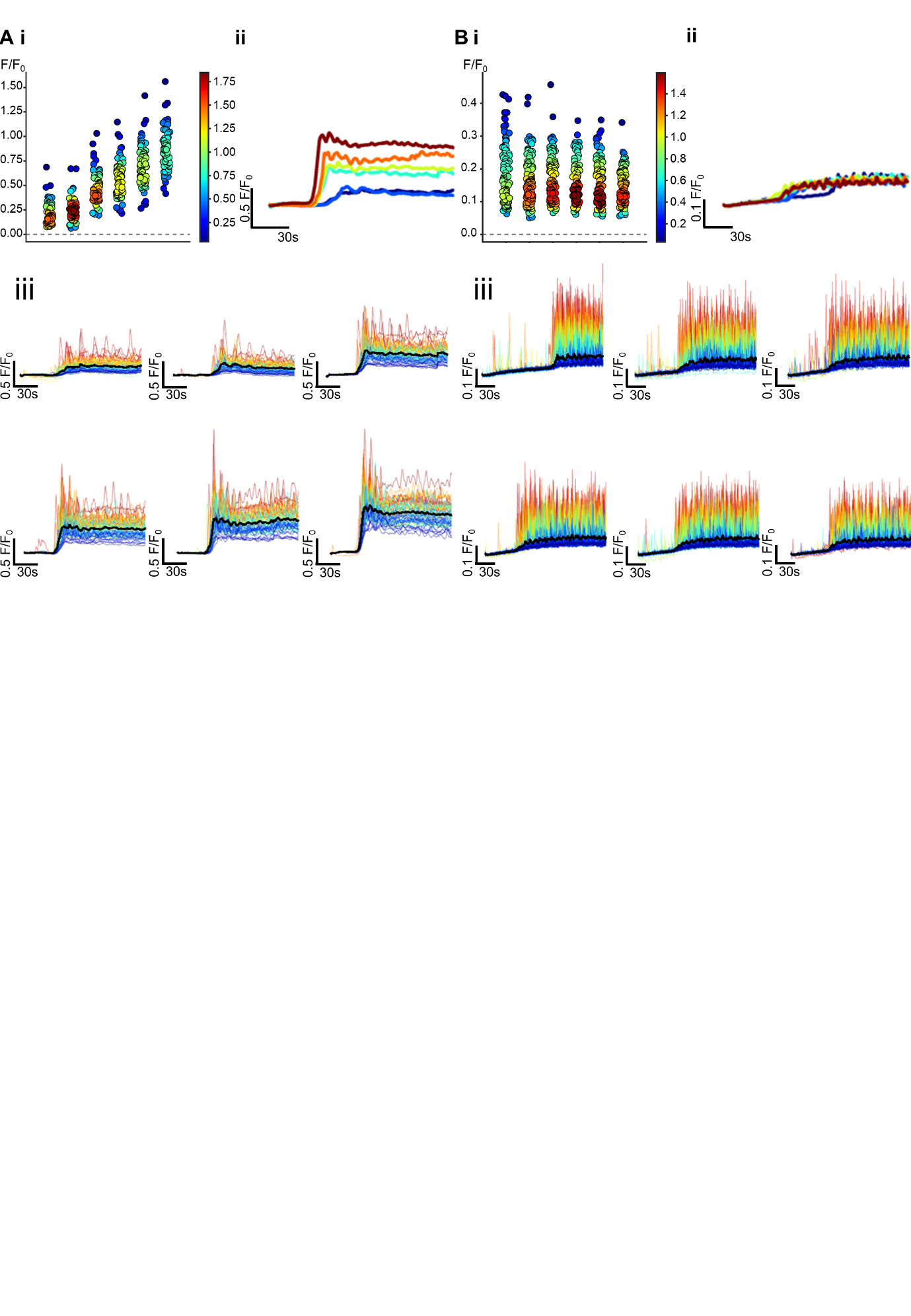


**Supplementary Figure 1: Repeated ACh administration to mesenteric endothelial cells leads to an increase in signal (‘run-up’) unless appropriate washes are performed**

Sequential addition of ACh (50 nM) to endothelial cells of *en face* mesenteric arterial preparations (15 mins apart, 6 repeats) either (A) immediately after mounting the artery or (B) after a 15 mins ACh (50 nM) wash followed by a 15 mins PSS wash, repeated twice (1.5 ml min^-1^). Each repeat was taken 15 mins apart. (i) Summary data of the average *F/F_0_* value from each cell for each repeat. (ii) The average signal across all cells for each repeat. Each colour represents a different repeat. (iii) Individual *F/F_0_* Ca^2+^ traces from each cell, for each ACh Application. Traces are coloured according to the intensity of the response from red to blue, and the average signal is overlaid in black.


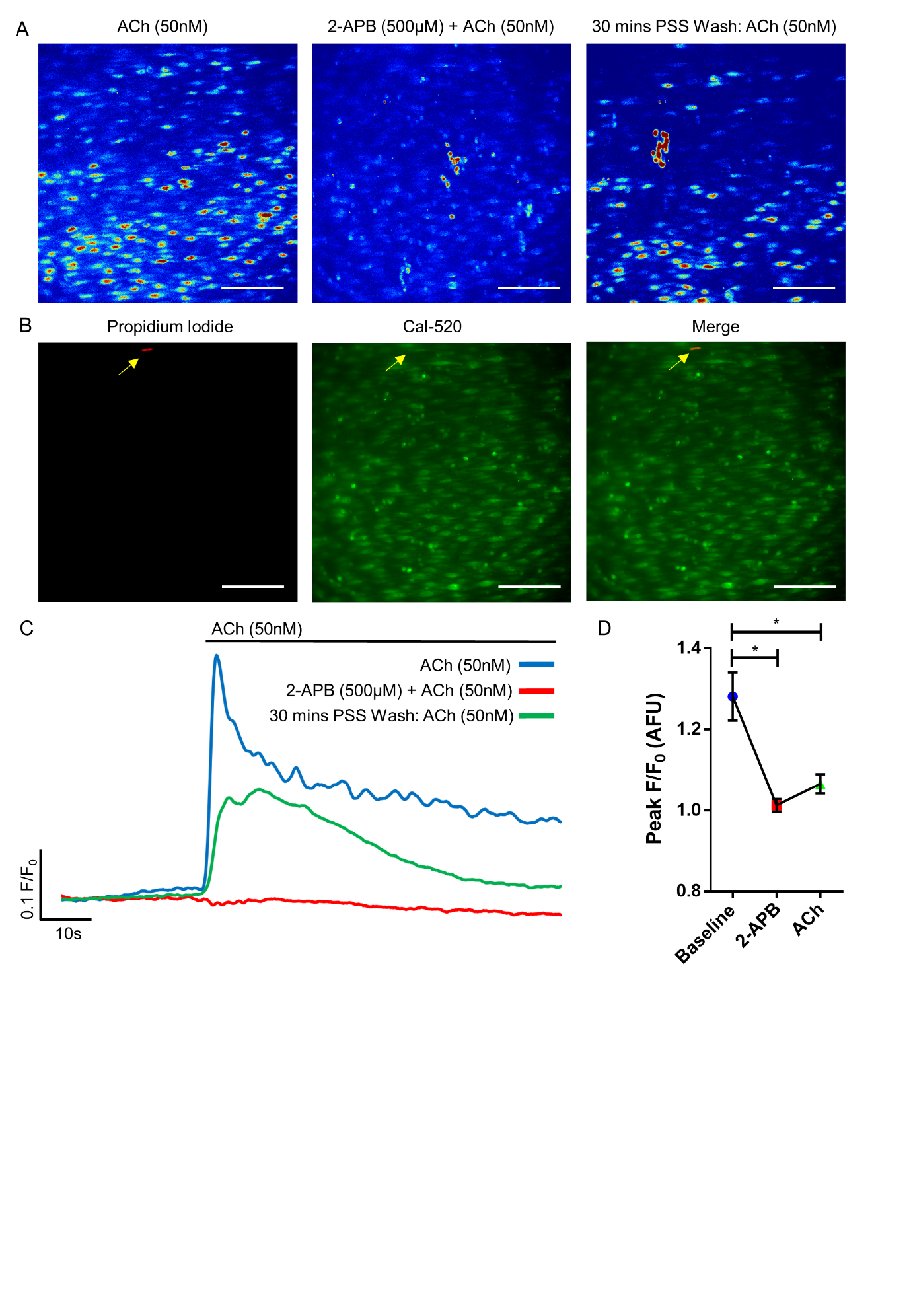


**Supplementary Figure 2: Endothelial function is partially recoverable after 2-APB**

Ca^2+^ release was stimulated via ACh (50 nM) under flow conditions. (A) Average intensity contrast-matched images (JET LUT) of the same endothelial cell Ca^2+^ levels after ACh stimulation at baseline, after 2-APB incubation or after a 30 mins PSS wash. Scale bar = 100 μm. (B) Propidium iodide (PI, 1.5 μM, 2 mins) shows that incubation with 2-APB (500 μM, 10 mins) did not cause toxicity in endothelial cells. Images are an average intensity projection of 100 images, shown as the PI channel, the Cal-520 channel and a merge. (C) Representative individual *F/F_0_* traces from ACh stimulation at baseline (blue), after 2-APB (500 μM, 10 mins. Red) and after 30 mins wash with PSS (green). Bar above trace indicates the ACh administration period. (D) Peak *F/F_0_* signals averaged across the field of view for each biological repeat, compared between baseline (blue), 2-APB incubation (red) and after 30 mins PSS wash (green) conditions. *n* = 5, *: *p* < 0.05.

**
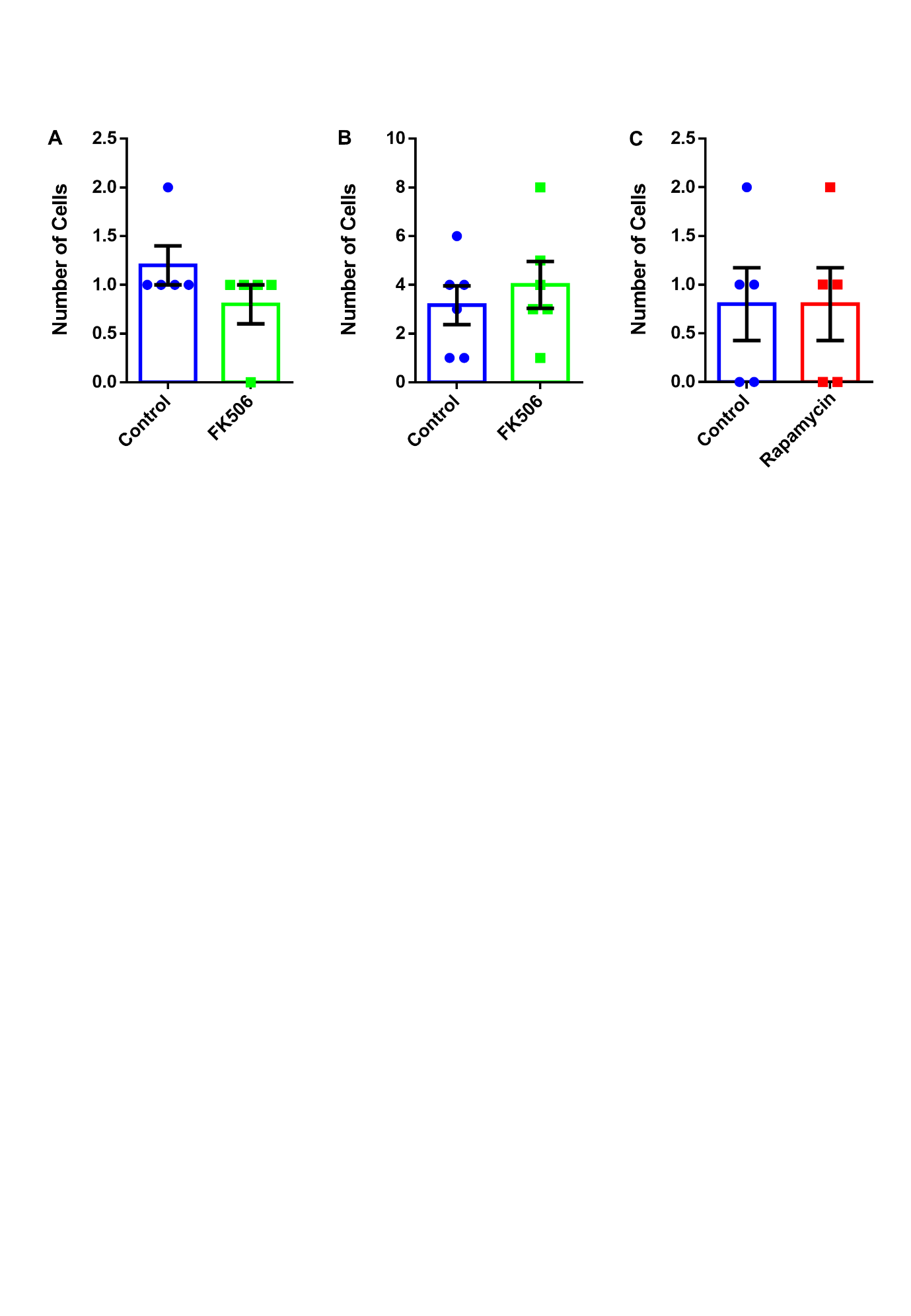
Supplementary Figure 3: FK506 or rapamycin did not cause Ca^2+^ leak**

The number of cells showing basal Ca^2+^ activity prior to stimulation were counted. All preparations were paired between control and FK506 or Rapamycin groups. (A) No flow conditions, FK506 (10 μM, 30 mins). (B) Flow conditions (1.5 ml min^-1^), FK506 (10 μM, 30 mins). (C) No flow conditions, rapamycin (10 μM, 30 mins). *n* = 5 ; * *p* < 0.05.

**Supplementary Video 1: ACh-evoked Ca^2+^ signals in *en face* mesenteric arterial endothelial cells**

Representative video of Ca^2+^ signals evoked by ACh (50 nM) flow (1.5 ml min^-1^) after ~60 s of baseline imaging. Images are shown as the raw grayscale Cal-520 (5 µM) image (left hand side) and with the fire LUT applied (right hand side) for improved contrast. Images taken at 10fps, scale bar = 50 µm.

**Supplementary Video 2: IP_3_-evoked Ca^2+^ signals in *en face* mesenteric arterial endothelial cells**

Representative video of localised caged-IP_3_ photolysis and the resulting Ca^2+^ signals, activated after 15 s of baseline imaging. Images are shown as the raw grayscale Cal-520 (5 µM) image (left hand side) and with the fire LUT applied (right hand side) for improved contrast. Images taken at 10fps, scale bar = 50 µm.

**Supplementary Video 3: Caffeine does not evoke Ca^2+^ signals in *en face* mesenteric arterial endothelial cells**

Representative video of endothelial Ca^2+^ signal during addition of caffeine (10 mM) after ~60 s of baseline imaging. Image is shown as the raw grayscale Cal-520 (5 µM) signal, with images taken at 10fps.

**Supplementary Video 4: ACh- and caffeine-evoked Ca^2+^ signal responses in dissociated aortic endothelial cell patches**

Representative video of Ca^2+^ signals evoked by gravity-driven perfusion of PSS (500s), ACh (50 nM, 500s) and caffeine (10 mM, 500s). Image sequence is shown as the raw grayscale Cal-520 (5 µM), with all the images contrast level-matched. Images taken at 10fps, scale bar = 10 µm.
